# Precocious Acquisition of Neuroepithelial Character in the Eye Field Underlies the Onset of Eye Morphogenesis

**DOI:** 10.1016/j.devcel.2013.09.023

**Published:** 2013-11-11

**Authors:** Kenzo Ivanovitch, Florencia Cavodeassi, Stephen W. Wilson

**Affiliations:** 1 Department of Cell and Developmental Biology, University College London, Gower Street, London WC1E 6BT, UK; 2 MRC Laboratory for Molecular Cell Biology, University College London, Gower Street, London WC1E 6BT, UK

## Abstract

Using high-resolution live imaging in zebrafish, we show that presumptive eye cells acquire apicobasal polarity and adopt neuroepithelial character prior to other regions of the neural plate. Neuroepithelial organization is first apparent at the margin of the eye field, whereas cells at its core have mesenchymal morphology. These core cells subsequently intercalate between the marginal cells contributing to the bilateral expansion of the optic vesicles. During later evagination, optic vesicle cells shorten, drawing their apical surfaces laterally relative to the basal lamina, resulting in further laterally directed evagination. The early neuroepithelial organization of the eye field requires Laminin1, and ectopic Laminin1 can redirect the apicobasal orientation of eye field cells. Furthermore, disrupting cell polarity through combined abrogation of the polarity protein Pard6γb and Laminin1 severely compromises optic vesicle evagination. Our studies elucidate the cellular events underlying early eye morphogenesis and provide a framework for understanding epithelialization and complex tissue formation.

## Highlights

• Live imaging reveals cellular behaviors during optic vesicle evagination
• Eye field cells acquire apicobasal polarization prior to other neural cells
• Laminin1 is required for the epithelial organization of basal eye field cells
• Mesenchymal-like core eye field cells intercalate into the basal epithelium

## Teaser

By performing high-resolution live-imaging experiments in zebrafish, Ivanovitch et al. resolve the dynamic cell behaviors underlying optic vesicle formation. The first event in this process is Laminin1-dependent epithelialization of cells at the margin of the eye field. Subsequent epithelial remodeling and cell intercalation events shape the evaginating optic vesicles.

## Introduction

One of the most common morphogenetic tissue reorganizations during embryonic development is epithelial evagination, a process that involves extensive changes in cell shape and behavior (Fristrom, 1988; Keller and Shook, 2011; Sawyer et al., 2010). One such evagination occurs as the eyes form. During early phases of nervous system development, the anterior neural plate (ANP) folds in such a way that two pouches evaginate from the lateral walls of the diencephalon to give rise to the optic vesicles, the primordia of the eyes. Early studies of this process in mouse and chick were based on analysis of fixed samples (Camatini and Ranzi, 1976; Svoboda and O’Shea, 1987) and suggested that the epithelial remodeling occurring during optic vesicle formation was promoted by changes in cell shape similar to those observed during other evagination events (Fristrom, 1988; Keller and Shook, 2011; Sawyer et al., 2010). More recently, technical advances in live imaging coupled with the optical accessibility of teleost embryos have facilitated analysis of optic vesicle formation in vivo (Cavodeassi et al., 2013; England et al., 2006; Kwan et al., 2012; Picker et al., 2009; Rembold et al., 2006), and such studies have led to novel insights into the cell behaviors accompanying eye development. Recent studies have shown that embryonic stem cells (ESCs) can aggregate and form optic vesicles in culture (Eiraku et al., 2011; Nakano et al., 2012), providing another model system for studying the processes underlying morphogenesis of the eyes. As yet, however, in none of these systems do we have a comprehensive understanding of the cell behaviors that lead to outpocketing of the eyes from the diencephalon, whereas adjacent forebrain domains show no comparable evagination.

The neural precursors destined to form the eyes occupy a single domain, the eye field, that spans the midline of the ANP. A previous live-imaging study in zebrafish suggested that folding of a coherent sheet of epithelial cells may underlie optic vesicle evagination (England et al., 2006), but as this study tracked the movement of cell nuclei, it did not allow assessment of the changes in cellular morphology or organization associated with evagination. Rather than being static structures, epithelia exhibit highly dynamic movements, and exchange of cell neighbors is commonly observed through live imaging of epithelial cells in other morphogenetic contexts, such as during germ band extension in *Drosophila* (Bertet et al., 2004; Butler et al., 2009; Guillot and Lecuit, 2013). Thus, it is plausible that eye field cells could form a coherent, polarized tissue with apical and basolateral surfaces and still show dynamic individual cell behaviors during morphogenesis. Indeed, in a different teleost, medaka, individual eye cells seem to be migratory during optic vesicle evagination (Rembold et al., 2006), suggesting that eye cells only acquire full neuroepithelial character at later stages of eye morphogenesis (Sinn and Wittbrodt, 2013).

As in the teleost eye field, epithelialization must be coupled with morphogenesis during formation of optic vesicles from in-vitro-cultured mouse and human ESCs (Eiraku et al., 2011; Nakano et al., 2012). A key requisite for the formation of optic vesicles in vitro is the culturing of aggregated cells in three-dimensional (3D) Matrigel, a major component of which is the extracellular matrix (ECM) protein Laminin1. This suggests that a basal lamina-like structure and perhaps apicobasal (AB) polarization of prospective eye cells facilitate the process of evagination.

In this study, we use high-resolution 4D confocal imaging to analyze some of the key cellular events and behaviors that underlie optic vesicle evagination in zebrafish. Through mosaic labeling of single cells and subcellular components combined with cell tracking over time, we find that the eye field is constituted by two discrete populations of cells. Basally positioned cells acquire AB polarity and establish a pseudostratified neuroepithelial organization prior to the onset of optic vesicle evagination and precociously as compared to neighboring neural tissues. This process is instructed by the presence of a Laminin-rich basal lamina and accompanies the initial bulging out of the eye field. In the absence of Laminin, basal cells lose their coordinated AB polarity, fail to properly elongate, and evagination is consequently compromised. The second population of cells is located at the apical core of the eye field, and these cells remain mesenchymal as the basal cells epithelialize. At later stages, these cells undergo a mesenchymal-to-epithelial transformation (MET) during which they elongate and intercalate between cells of the basal, epithelialized domain of the eye field thereby contributing to the later steps of tissue evagination. Confirming a requirement for epithelialization and coordination of AB polarity during early morphogenesis of the eye, combined disruption of the polarity protein Pard6γb and Laminin1 compromises optic vesicle evagination.

## Results

### The ANP Undergoes a Distinct Morphogenetic Reorganization as Compared to Other Regions of the Neural Plate

**Figure 1:**
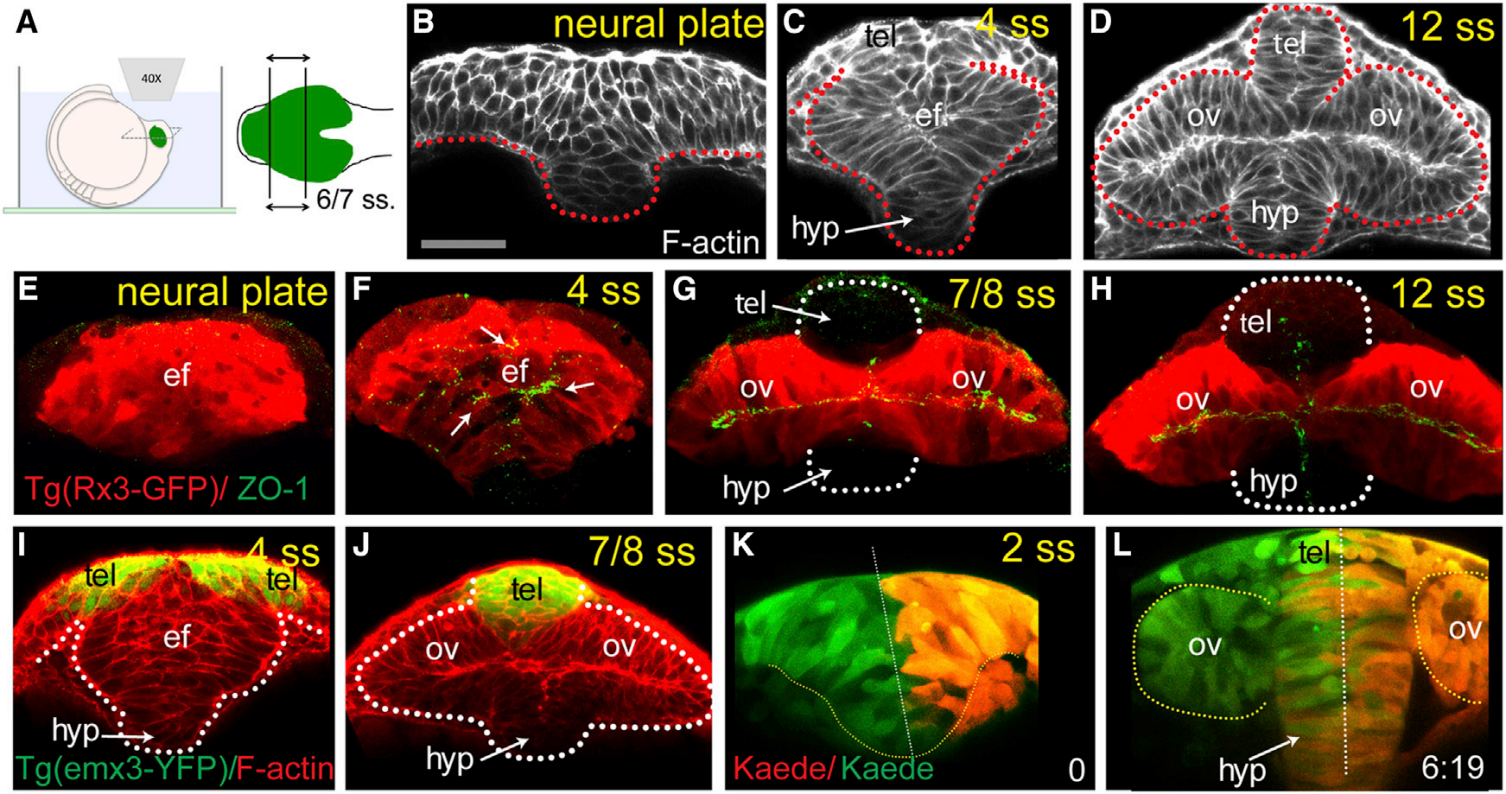
Reduced Convergence and Precocious AB Polarization of Eye Field Cells (A) These panels and those in other figures show transverse (frontal) confocal sections through the developing ANP at the level of the eye field/optic vesicles, imaged as schematized in (A). (B–D) Wild-type embryos stained with F-actin to reveal cellular organization. tel, telencephalon; ef, eye field; hyp, hypothalamus; ov, optic vesicle. Scale bar, 64 μm (B). (E–H) *Tg*(*rx3:gfp*) embryos immunostained against GFP (red) and ZO-1 (green/yellow, arrows in F) to reveal cell polarity. (I and J) *Tg*(*emx3:yfp*) embryos immunostained against GFP (green) and stained for F-actin (red), showing the reorganization of the telencephalon that accompanies optic vesicle evagination. (K and L) Initial (K) and final (L) time points of a time-lapse movie sequence of an embryo expressing Kaede. One-half of the neural plate was photoconverted from green to red by UV illumination (t = 0). White dotted lines in (G) and (H) outline the telencephalon and hypothalamus; red/white lines in (B)–(D) and (K) and (L) outline the neural anlage. See also Figure S1 and Movie S1.

Cell behaviors underlying neurulation at the level of the ANP are likely to be distinct from those in other regions of the neural plate because, in this domain, cells comprising the eye field evaginate to give rise to bilateral optic vesicles concomitant with neural tube formation. To determine if this is indeed the case, we compared the organization and behavior of eye field cells to that of other neural plate territories (e.g., Tawk et al., 2007). First, to assess the changes in cellular organization of the ANP that occur during neurulation, we performed confocal imaging of transverse views of whole embryos (Figure 1A) from neural plate stage (10.5 hr postfertilization [hpf]) to 12 somite stage ([ss], 15 hpf), by which stage the optic vesicles have emerged laterally from the walls of the forming forebrain.

As also observed by Rembold et al. (2006), eye field cells show reduced medio-lateral convergence (Figures 1B, 1C, 1E, and 1F) and never form the compact neural keel characteristic of more caudal neural plate regions (Tawk et al., 2007). Consequently, the optic vesicles form lateral bulges as the keel forms (Figures 1C and 1F). Reduced convergence leaves a gap at the dorsal midline of the ANP, and as the lateral edges of the forming optic vesicles roll up, this gap is filled with eye field cells (Figure 1C). The eye field subsequently resolves into bilateral optic vesicles (Figures 1D, 1G, and 1H), whereas other regions of the neural plate, including the more dorsally located telencephalon (Figures 1G–1J), further converge to form a solid neural rod (reviewed in Clarke, 2009).

A characteristic feature of neural keel formation is that upon reaching the midline, neuroepithelial cells undergo midline-crossing divisions (c-divisions) to distribute siblings on each side of the neural tube (Ciruna et al., 2006; Geldmacher-Voss et al., 2003; Kimmel et al., 1994; Tawk et al., 2007). Neuroepithelial cells acquire AB polarity coincident with this c-division and as a consequence, organize as a polarized coherent epithelium (Buckley et al., 2013; Tawk et al., 2007; Žigman et al., 2011). To determine if eye field cells undergo similar crossing behaviors, we used the photoconvertible Kaede protein (Ando et al., 2002) to differentially label left and right sides of the ANP (Figure 1K; see Movie S1 available online).

As the optic vesicles evaginate, most eye field cells remain on the same side of the midline (Figures 1K and 1L), indicating that if present at all, c-divisions are rare within the forming optic vesicles. In contrast, within both the hypothalamus and the telencephalon, we observed intermixing of green and red cells on both left and right sides (Figure 1L), suggesting that c-divisions are common in these territories. The absence of midline crossing at the level of the eye field indicates that acquisition of AB polarity cannot be linked to c-divisions and must happen through alternative mechanisms in this domain. Consequently, we next analyzed where and when AB polarity arises in the ANP.

The acquisition of AB polarity by eye field cells occurs precociously as compared to cells in other neural plate domains. There are punctate accumulations of centrosomes and apical polarity markers such as the tight junction component zona-occludens-1 (ZO-1) and the polarity protein aPKC in central region of the eye field as early as 3–4 ss (Figures 1F and S1A–S1D), and a coherent apical midline domain is observed from 6 ss (Figures 1G and 1H; data not shown), coincident with the onset of optic vesicle evagination. At these stages, no overt polarization is detected in other regions of the neural plate (Geldmacher-Voss et al., 2003; Tawk et al., 2007). For instance, within the telencephalon, an apical domain is only evident by 12 ss (compare Figures 1G and 1H). Consistent with the early onset of AB polarization in the eye field, the apical polarity determinant *pard6γβ* is expressed exclusively in the eye field (Figure S1E) for several hours before expression is initiated in other regions of the neural plate (data not shown). Thus, the cellular mechanisms involved in neuroepithelial morphogenesis of the optic vesicles are distinct from those occurring during posterior neural tube formation. We next looked more directly at the cellular organization of the eye field.

### Two Morphologically Distinct Populations of Cells Arise within the Eye Field at the Onset of Optic Vesicle Morphogenesis

**Figure 2:**
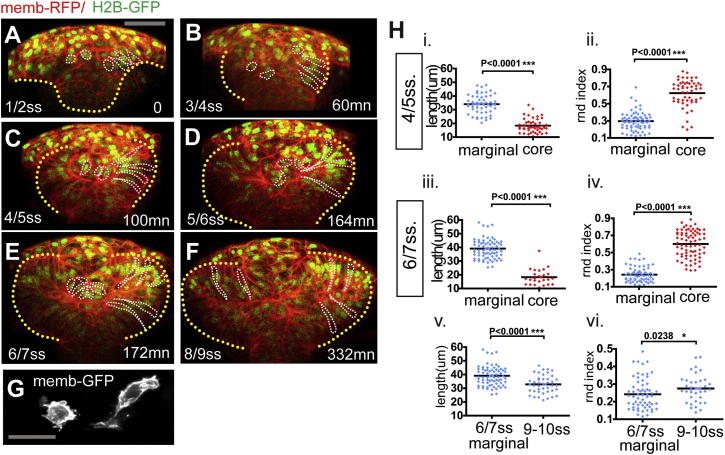
Two Morphologically Distinct Cell Populations within the Forming Optic Vesicles (A–F) Time-lapse movie from neural plate-1 ss (t = 0 min, 10.5 hpf) up to 8–9 ss (t = 332 min, around 13.5 hpf) of an embryo ubiquitously expressing membrane-RFP (red) and H2B-GFP (green). The yellow dotted line highlights the neural anlage at t = 0 (A) and the eye field/optic vesicles at subsequent time points. Example marginal and core cells are outlined in white (B–F). Detailed morphology is not evident at this magnification but can be seen in (G) and in other figures. Scale bar, 64 μm (A). (G) Membrane-GFP labeling of typical core and marginal cells at 4 ss. Scale bar, 27 μm. (H) Quantification of the cell length (i, iii, and v) and the cell rdn index (ii, iv, and vi) of marginal (blue) and core (red) cells. Black bars indicate mean (Mann-Whitney U test). Numbers of cells quantified are as follows: (i) cell length at 4–5 ss, n = 56 marginal cells in eight embryos and n = 48 core cells in five embryos; (ii) rdn index at 4–5 ss, n = 71 marginal cells in eight embryos and n = 62 core cells in five embryos; (iii) cell length at 6–7 ss, n = 73 marginal cells in eight embryos and n = 26 core cells in five embryos; (iv) rdn index at 6–7 ss, n = 63 marginal cells in eight embryos and n = 53 core cells in five embryos; (v) marginal cell length at 9–10 ss, n = 58 cells in five embryos; and (vi) marginal cell rdn at 9–10 ss, n = 38 cells in four embryos. In order to distinguish unambiguously the shape of single eye field cells, in three-dimensions, large z stacks (60 μm in average) were acquired on mosaically labeled whole-mount embryos. See also Movie S2.

To resolve the changes in cellular organization that accompany optic vesicle evagination, we carried out live confocal imaging at high temporal and spatial resolution of embryos expressing membrane- and nuclear-localized red fluorescent protein (RFP) and GFP (five movies, each over a period of 300 min; Figures 2A–2F; Movie S2).

Two morphologically distinct populations of cells arise within the eye field prior to the onset of optic vesicle evagination. At 1 ss (t = 0; Figure 2A), there is no overt cellular organization within the ANP, but from 3 ss onward (t = 60 min, Figure 2B), the anlage of the eye field rounds up, and those cells located at its basal surface adopt a slightly elongated appearance and radial orientation. The cells located at the core of the eye field instead remain disorganized (Figure 2B). Between 4–5 ss (t = 100 min) and 6–7 ss (t = 174 min), the two morphologically distinct populations of eye field cells are more clearly distinguishable (Figures 2C–2E, 2G, and 2H). The cells located at the edge of the eye field become radially elongated, forming a contiguous layer of cells (“marginal cells”; cell length is 34.05 ± 6.7 μm at 4–5 ss increasing to 39.09 ± 7.7 μm at 6–7 ss, mean ± SD, p = 0.006; roundness [rdn] index at 4–5 ss is 0.30 ± 0.10 and at 6–7 ss is 0.24 ± 0.09, mean ± SD, p = 0.002; Figure 2H). Marginal eye field cells surround a mass of disorganized and more rounded eye field cells (“core cells”; cell length at 4–5 ss is 18.29 ± 1.1 μm and at 6–7 ss is 18.36 μm ± 0.8, mean ± SD, p = 0.83; rdn index at 4–5 ss is 0.60 ± 1.17 and at 6–7 ss is 0.62 ± 0.17, mean ± SD, p = 0.66; Figure 2H). Core cells do not elongate during this period (Figures 2B–2D), and their appearance is not associated with mitotic events (Movie S2; data not shown). At 4–5 ss, core cells comprise 33% ± 7.1% of the total number of eye field cells (n = 7 embryos, mean ± SD), and this reduces to 21% ± 2.7% by 6–7 ss (n = 5 embryos, mean ± SD). Thus, whereas cells in other regions of the neural plate elongate medio-laterally in a coordinated way at late stages of neurulation (Geldmacher-Voss et al., 2003; Hong et al., 2010), eye field cells organize and elongate precociously but only if located at the margin of the eye field domain. The core of the eye field remains filled with cells of mesenchymal morphology.

### Marginal Eye Field Cells Adopt Neuroepithelial Organization at the Onset of Eye Morphogenesis

**Figure 3:**
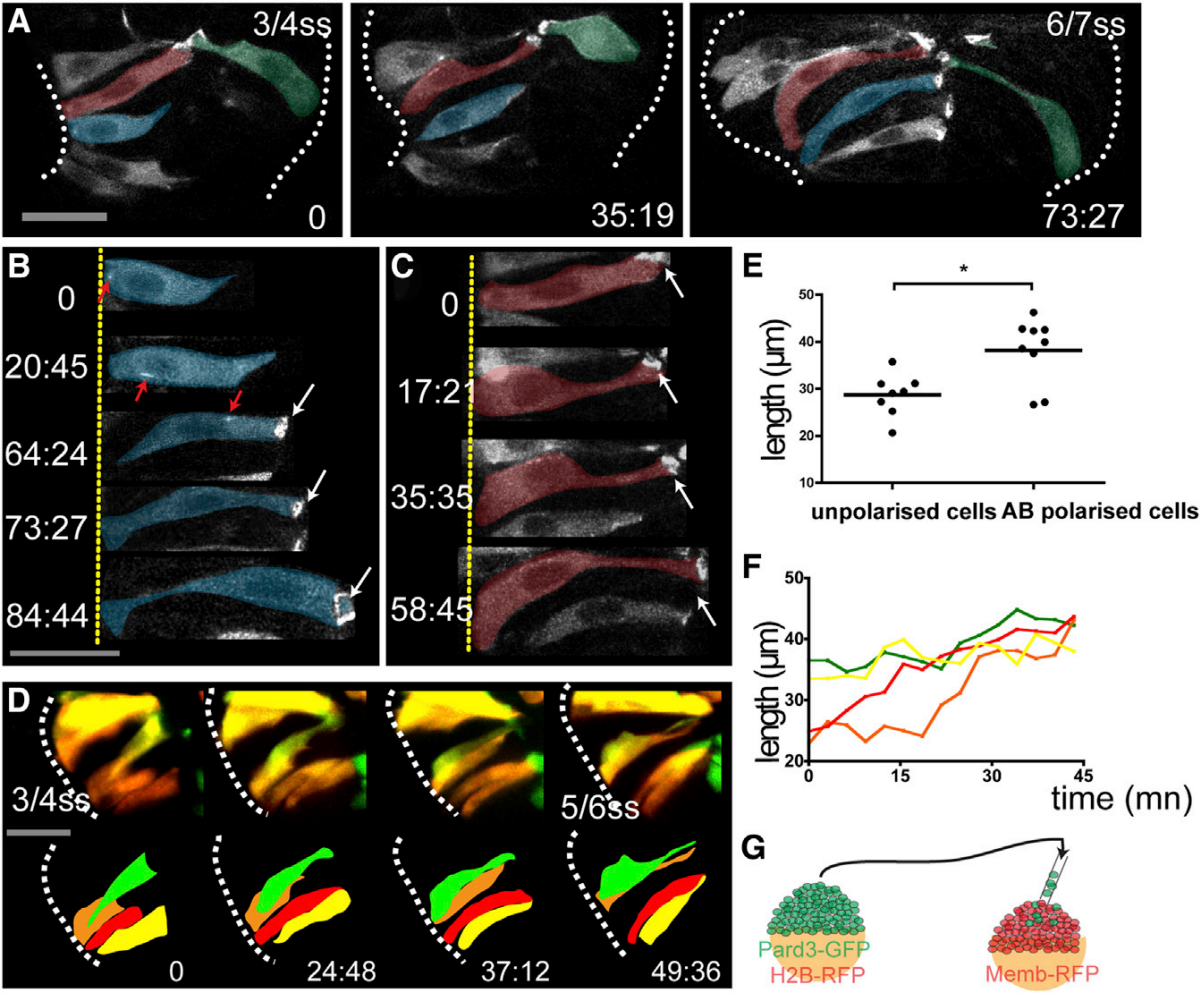
Marginal Eye Field Cells Acquire AB Polarity in a Coordinated Manner (A) Time-lapse movie of cells in the eye field of an embryo mosaically expressing Pard3-GFP. Cells are pseudocolored for clarity. (B and C) High-resolution images of two marginal cells extracted from the movie in (A) as they polarize. Red arrows in (B) show Pard3-GFP punctae as they are transported apically. White arrows in (B) and (C) show apical accumulation of Pard3-GFP. (D) Time-lapse movie of cells in the eye field of an embryo mosaically labeled with Kaede. Single cells were photoconverted from green to red by illumination with UV light. Four adjacent marginal cells are followed as they gradually coordinate their length. (E) Unpolarized marginal cells are shorter than the polarized ones (black bar indicates mean [Mann-Whitney U test]; n = 30 from five embryos). (F) Quantification of cell length of the four adjacent marginal cells in (D) over time (cell length is measured every 4.50 min). (G) Schematic showing the experimental strategy. White dotted lines (A) and (D) outline the eye field. Scale bars, 27 μm (A, B, and D). See also Figure S2 and Movie S3.

The organization of the marginal layer suggests the precocious establishment of an AB-polarized neuroepithelium around the basal surface of the evaginating optic vesicles. To address whether this is the case, we mosaically expressed a GFP-tagged version of Pard3, a polarity protein involved in the establishment of epithelial AB polarity in both vertebrates and invertebrates (Alexandre et al., 2010; Laprise and Tepass, 2011; von Trotha et al., 2006) (Figure 3G), and tracked AB polarization and shape changes of individual eye field cells.

At 3–4 ss, marginal cells are not organized as an AB-polarized pseudostratified neuroepithelium because they are heterogeneous both in shape/length and expression of polarity markers. Indeed, neighboring marginal cells have irregular and uncoordinated cell length, and although many accumulate polarized Pard3-GFP (70%, n = 30 marginal cells in five embryos; Figure 3A), some do not. Moreover, marginal cells lacking polarized Pard3-GFP are significantly shorter than those that are polarized (28.7 ± 1.6 μm versus 38.1 ± 2.3 μm, p = 0.02, mean ± SD; Figure 3E).

During subsequent stages of morphogenesis, marginal cells rearrange as a pseudostratified neuroepithelium by gradually elongating and coordinating their length with their neighbors (Figures 3D and 3F). Cell elongation coincides with the establishment of the apical domain, and Pard3-GFP localization can acquire a ring-like pattern suggestive of localization at adherens junctions (Alexandre et al., 2010; Figures 3B and 3C; Movie S3). By 6 ss, 95% of the marginal eye field cells are polarized and organized as a pseudostratified monolayer of cells (Figure 3A, 73:27). As marginal cells divide, their nuclei relocate apically, and daughter cells reintegrate in the marginal layer (n = 7 out of 7 in four embryos; Movie S4), a behavior typical of neuroepithelial cells undergoing interkinetic nuclear movements (IKNMs). Throughout this whole process, marginal cells remain attached to the basal surface as the eye field initiates the large-scale morphogenetic reorganization that forms the optic vesicles.

These results show that localization of Pard3 in marginal eye field cells occurs at very early morphogenetic stages and is not preceded by a c-division, contrasting with the situation in more posterior regions of the forming neural tube (Buckley et al., 2013; Tawk et al., 2007). In the telencephalon, however, neuroepithelial cells acquire AB polarity at later stages as compared to eye field cells, in a manner more similar to the hindbrain. Moreover, at least in some cases, this polarization follows a c-division (n = 7 out of 10 in four embryos; Figure S2).

### Core Eye Field Cells Localize Pard3-GFP in a Polarized Manner

**Figure 4:**
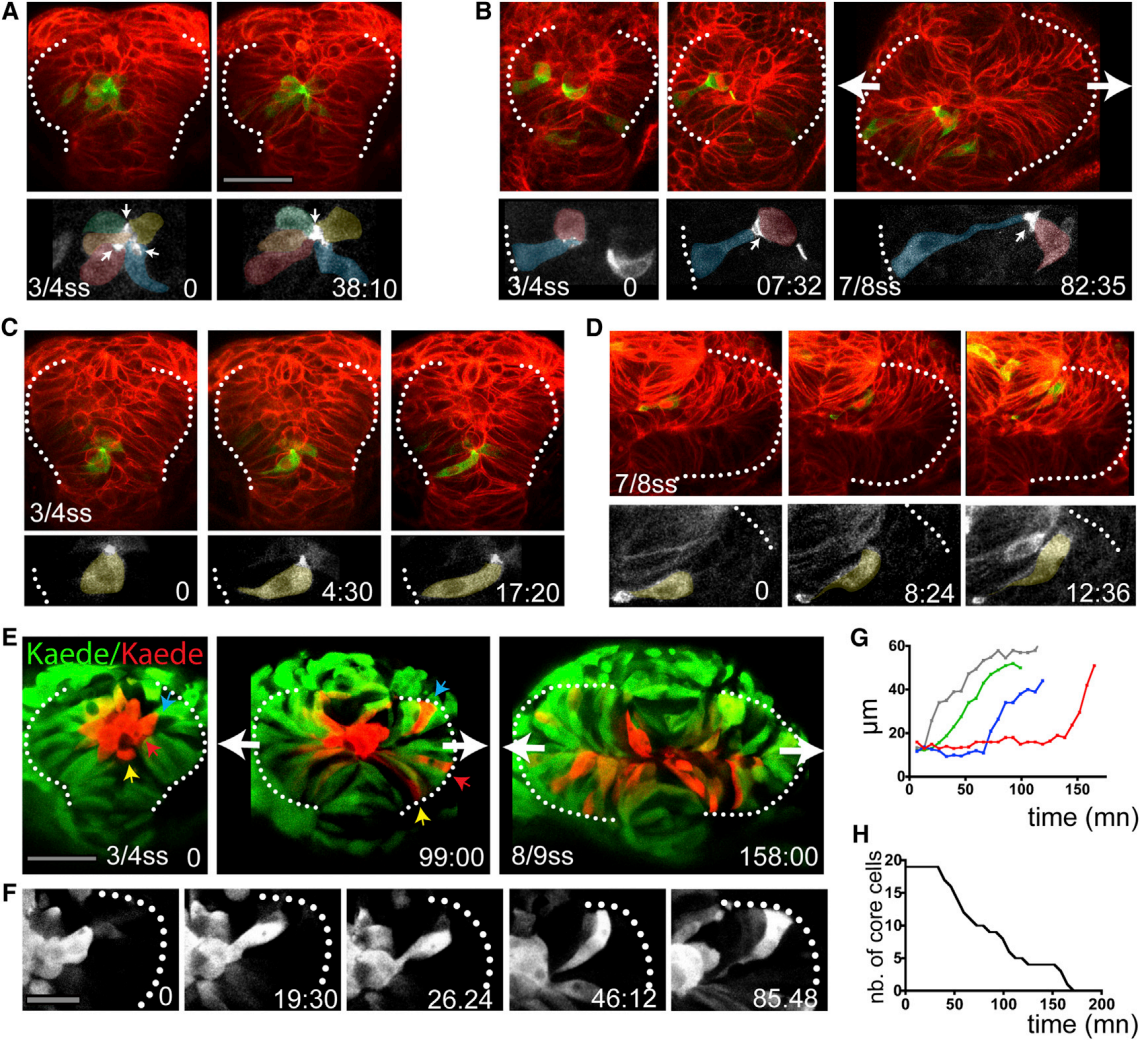
Core Eye Field Cells Polarize and Intercalate in the Marginal Layer during Eye Morphogenesis (A–D) Time-lapse movies of eye field core cells expressing the fusion protein Pard3-GFP (green). Embryos are counterlabeled with a membrane-RFP (red). Insets below show the green channel only (Pard3-GFP). (E) Time-lapse movie of an embryo expressing Kaede. Core cells were labeled in red by photoconversion of Kaede from green to red. Colored arrows point at individual core cells as they intercalate within the optic vesicles. (F) Close-up of a single core cell elongating and acquiring a wedge shape as it intercalates. (G) Quantification of cell length over time in individual core cells with data extracted from the movie sequence in (E). (H) Graph showing the reduction in the number of core cells as morphogenesis proceeds (18 core cells were tracked from t = 0 onward). White dotted lines in (A)–(F) outline the eye field. Lateral arrows in (B) and (E) indicate the direction of evagination of the tissue. Scale bars, 64 μm (A and E) and 27 μm (F). See also Figure S3 and Movies S4, S5, S6, S7, and S8.

Cells in the core of the eye field are not in contact with the basal lamina and are mesenchymal in morphology (Figure 2G). Despite this, core cells also show polarized accumulation of Pard3-GFP (Figures 4A–4D; 78%, n = 18 at 4 ss in six embryos; Movie S4). Polarized localization of Pard3-GFP in core cells is initially not overtly correlated with any specific position within the cell cortex. Nevertheless, neighboring core cells can coordinate Pard3-GFP localization when organized as rosette-like structures (Figures 4A and S3; Movie S5; seen in four embryos). In some other cases, Pard3-GFP accumulated and stabilized at the contact point between a core cell and the apical surface of a marginal cell (Figure 4B; Movie S6; n = 4 in six embryos). Thus, the absence of contact of the core cells with the basal lamina does not prevent them from localizing polarity proteins in a polarized manner.

### Core Eye Field Cells Integrate in the Marginal Eye Field Layer by Intercalation

The mesenchymal morphology of core cells is reminiscent of that of the migratory eye field cells described by Rembold et al. (2006) that are proposed to drive optic vesicle evagination. To assess whether core cells do migrate and how they relate to the process of evagination, we labeled them by photoconverting Kaede in the core region of the eye field and tracked them from 4 ss until the completion of optic vesicle evagination.

Core cells integrate into the nascent epithelium of the forming optic vesicles by intercalation between marginal cells (Figures 4E and 4F; Movie S7). Core cells pass through an initial phase during which they remain at the midline, and their length is stable (rate of elongation is 0.0084 ± 0.00 μm/min^−1^, mean ± SD, n = 8 in five embryos with individual cells tracked between 60 and 149 min; Figure 4G). Subsequently, their somata undergo a fast elongation phase as they intercalate into the evaginating optic vesicles (rate of elongation is 0.78 ± 0.46 μm/min^−1^, mean ± SD, n = 15 in four embryos; timing of intercalation from 21:42 min up to 40:18 min; Figures 4C–4G). During intercalation, the apical domains of the core cells remain anchored at the midline, and basal processes extend between marginal cells to reach the basal lamina (Figures 4C and 4D, n = 9 in seven embryos; Movie S8).

Core cells intercalate gradually and stochastically (Figures 4E, 4G, and 4H), concurrent with the evagination of the eye field into optic vesicles. Consequently, the first core cells to intercalate tend to incorporate into the more distal region of the optic vesicles, and as evagination proceeds, intercalating cells gradually populate more proximal regions. Core cells are thus not predetermined to populate any particular region within the optic vesicles or, presumably, to acquire any particular retinal fate. Instead, once integrated in the optic vesicle, core cells become indistinguishable from the marginal eye field cells.

### Eye Field Cells Contract Their Apical Processes as the Optic Vesicles Evaginate

The pseudostratified neuroepithelium of the evaginating optic vesicles is constituted by the precociously AB-polarized marginal cells and the core cells that intercalate between them. To visualize any further changes in cellular organization that accompany later steps in evagination of the optic vesicles, we imaged individual cells from 7–8 ss to the completion of evagination.

**Figure 5:**
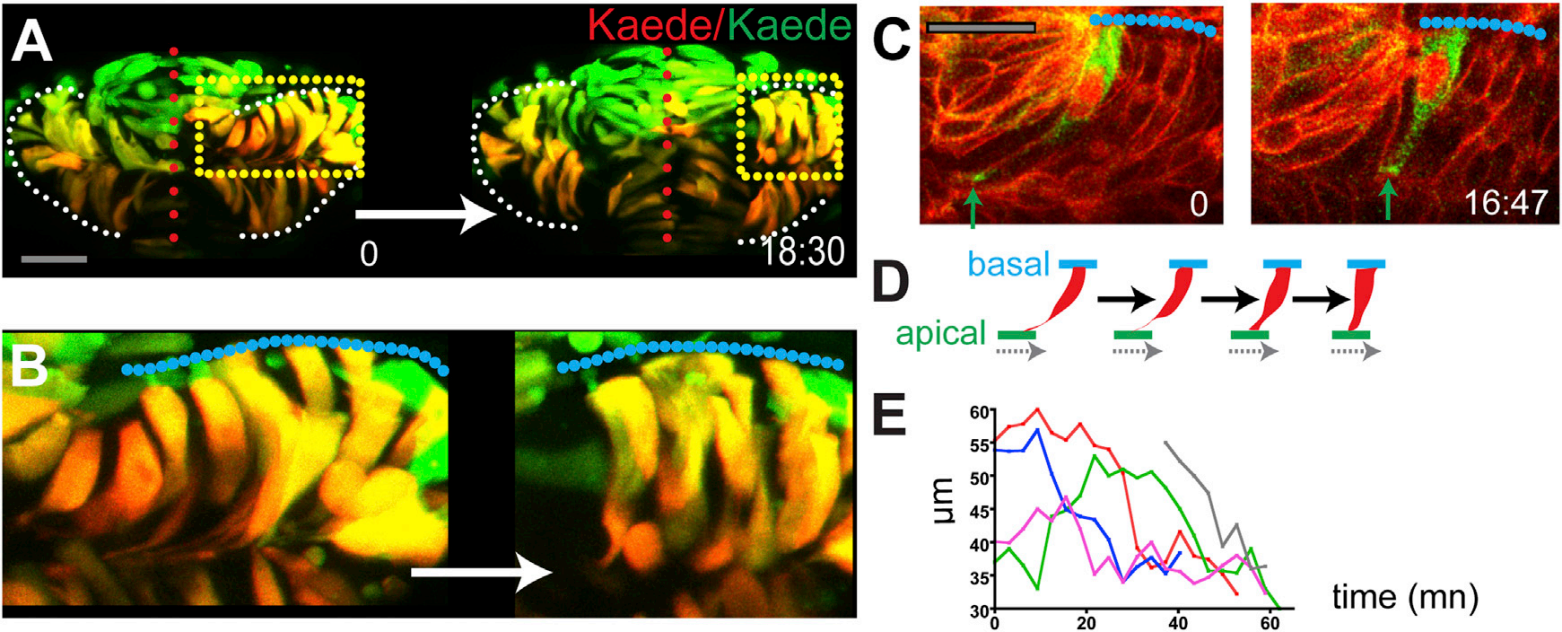
Cells Shorten at Late Stages of Optic Vesicle Evagination (A and B) Time-lapse movie starting at around 8–9 ss (z stack projection, six z slices, 5 μm intervals) of evaginating optic vesicles in an embryo mosaically labeled with Kaede. (C) Time-lapse movie starting at around 8–9 ss of a cell expressing the fusion protein Pard3-GFP in the eye field of an embryo ubiquitously expressing a membrane localized RFP fusion protein, as it shortens (green arrows). (D) Schematic of the labeled cell in (C). (E) Quantification of cell length over time (five cells were followed from around 6–7 ss in two embryos; cell length was measured at around every 5–6 min). White (A) and blue (B and C) dotted lines outline the edge of the optic vesicles, red dotted lines in (A) highlight the midline of the embryo, and yellow dotted boxes in (A) correspond to the regions shown in (B). Scale bars, 64 μm (A) and 27 μm (C).

During late stages of evagination, cells contract their apical processes and shorten as they move distally within the optic vesicles (Figure 5). At 7–8 ss, optic vesicle cells extend long apical processes and are radially oriented (Figures 5A and 5B, t = 0), with the exception of some cells in the most distal portion of the optic vesicles, which are shorter (data not shown). As the optic vesicles further evaginate, eye field cells shorten along their AB axis by contracting their apical processes and coincidently transition to a more dorsoventral orientation (cell length at 6–7 ss is 39.09 ± 7.7 μm and decreasing at 9–10 ss to 32.8 ± 6.5 μm, mean ± SD, p < 0.001; rdn index at 6–7 ss is 0.24 ± 0.09 and increasing at 9–10 ss to 0.28 ± 0.19, mean ± SD, p = 0.0238, Figure 2H; average rate of cell retraction for the cells shown in Figure 5E is −0.63 ± 0.22 μm/min^−1^, mean ± SD, Figures 5C–5E), resulting in the lateral displacement of their apical surface relative to their basal surface.

### Laminin1 Coordinates AB Organization of the Marginal Eye Field

Laminin1 is essential for the formation of epithelial structures in various in vitro and in vivo contexts (reviewed in Yurchenco, 2011). Given that marginal eye field cells show precocious polarization and epithelial character compared to other regions of the nervous system, we determined if this is associated with the precocious establishment of a Laminin1-rich basal lamina and, if so, whether Laminin1 is required for the polarization of eye field cells.

**Figure 6:**
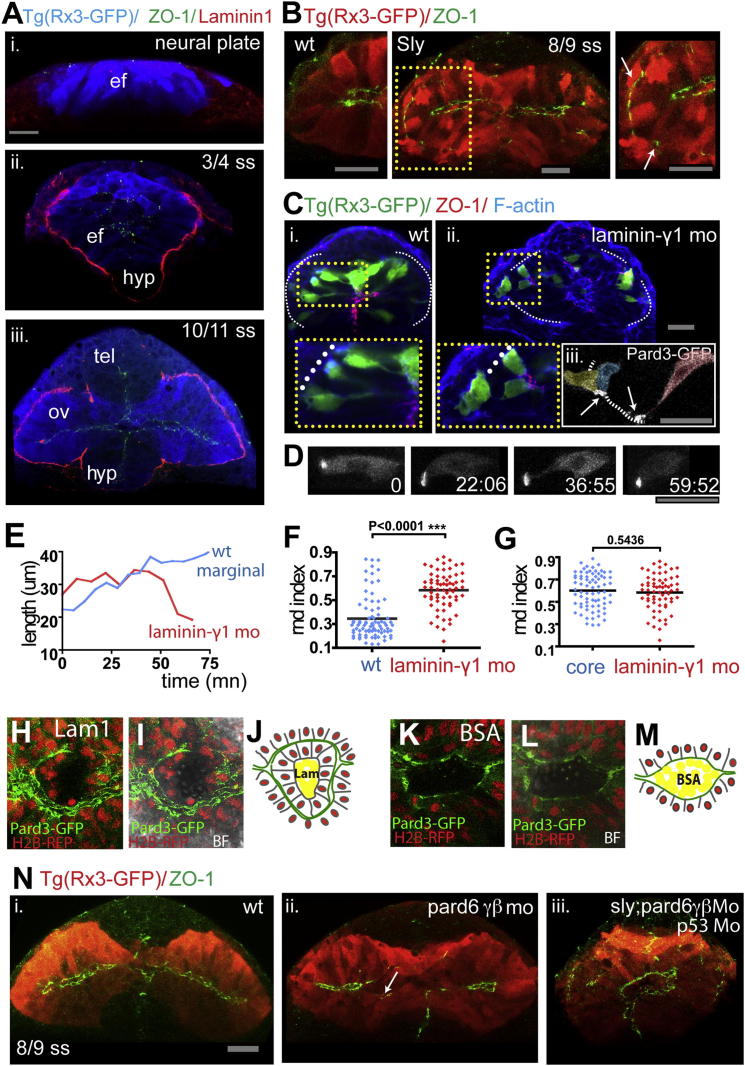
Laminin1 and Pard6γβ Are Required for Eye Morphogenesis Progression (A) Eye field and optic vesicles of fixed *Tg(rx3:gfp)* embryos immunostained against ZO-1 (green), Laminin1 (red), and GFP (blue). (B) *Tg(rx3:gfp)* and *Tg(rx3:gfp)/sly* optic vesicles immunostained against GFP (red) and ZO-1 (green). White arrows point to ZO-1 mis-localizing basally within the optic vesicles. (C) Transplants of *Tg*(*rx3:gfp*) expressing cells into nontransgenic hosts are shown: wild-type cells into wild-type embryos (Ci) and *lamininγ1* morphant cells into *lamininγ1* morphant embryos (Cii), immunostained against GFP (green), ZO-1 (red), and F-actin (blue), showing the disrupted epithelial organization (Cii) and the aberrant AB polarity axis (Ciii) in *lamininγ1* morphants. (D) Time-lapse movie of a *lamininγ1* morphant marginal cell expressing Pard3-GFP. (E) Changes in cell length over time of a wild-type (blue) and *lamininγ1* morphant (red) marginal cell transplanted into wild-type and morphant hosts, respectively. (F) Measurement of cell rdn index of 6–7 ss eye field cells in a wild-type (blue) and *lamininγ1* morphant embryo (red); *lamininγ1* morphant eye field cells are rounder than wild-type eye cells (wild-type, 0.34 ± 0.18; *lamininγ1* MO, 0.58 ± 0.18, mean ± SD). Black bar indicates mean (Mann-Whitney U test). WT, n = 83 cells in five embryos, Lamininγ1 mo n = 66 cells in four embryos, p < 0.0001. All eye field cells (marginal and core) have been included in the measurement. (G) *lamininγ1* morphant eye cells (presumptive core and marginal in red) are similar in their rdn index to core wild-type cells (blue). Black bar indicates mean (Mann-Whitney U test). WT core cells, n = 53 core cells in five embryos; Lamininγ1 morphant, n = 66 cells in four embryos, p = 0.5436. (H, I, K, and L) Images showing apical polarity organization of cells (labeled by Pard3-GFP, green) in the optic vesicles of embryos in which Laminin1-coated (H and I) or BSA-coated (K and L) beads were implanted in the center of the eye field. See also Movie S9. BF, bright field. (J and M) Schematics illustrating the organization of cells around beads in the eyes from (H) and (K). (N) *Tg(rx3:GFP)* (Ni), *Tg(rx3:GFP);pard6γβMo* (Nii), and *Tg(rx3:GFP);sly;pard6γβMo* (Niii) embryos stained as detailed in the figure. The arrow in (Nii) points at defective polarization in *pard6γβ* morphants. White lines in (C), (H), and (I) outline the margins of the eye field. Scale bars, 27 μm (A–D and N). See also Figure S4 and Movie S9.

Laminin1 is robustly enriched around the basal surface of the forming optic vesicles concomitant with the organization and polarization of marginal eye field cells (Figure 6A). In other regions, such as the forming telencephalon (Figure 6Aiii), Laminin1 immunoreactivity is much less robust than around the evaginating optic vesicles. To determine if Laminin1 acts as an external cue to organize marginal eye field cells, we analyzed the organization of the optic vesicles in the absence of Laminin1 following injection of morpholino oligonucleotides against Laminin-γ1, one of the three chains that make up Laminin1, or in *laminin-γ1* (*sleepy*, *sly*) mutants (Buckley et al., 2013; Dolez et al., 2011; Parsons et al., 2002; Peterson and Henry, 2010).

Marginal eye field cells of *sly* mutants and *laminin-γ1* morphants (Figures 6B, S4B, and S4C) show misoriented AB polarity and disrupted morphogenesis. Indeed, apical markers are present not only at the nascent apical surface of the evaginating optic vesicles but also at their basal surfaces. Mosaic labeling of cells in *laminin-γ1* morphants revealed that many marginally positioned eye field cells have a rounded morphology (comparable to the rounded morphology of core wild-type cells, Figure 6G; p = 0.5436), their axes of AB polarity can be aberrantly oriented (Figure 6Ciii), and sometimes even extend outside the eye field (rdn index >0.5 for 70% of the cells in the *laminin-γ1 MO*, rdn of cells in wild-type is 0.34 ± 0.18, and in *laminin-γ1 MO* is 0.58 ± 0.18, mean ± SD; p < 0.0001; Figures 6C and 6F).

Time-lapse analyses revealed that eye field cells have a very unstable cell shape in the absence of Laminin1. Eye field cells in morphants can elongate as in wild-types, but they can also occasionally retract (observed six times in six movies; Figures 6D and 6E), a process we never observed in wild-type embryos. Thus, Laminin1 is required not only for the initial coordinated polarization and organization of eye field cells but also for the maintenance of their neuroepithelial morphology. Laminin1 is required for these events non-cell-autonomously because the abnormal behavior of *laminin-γ1* morphant cells is rescued in a wild-type environment (Figure S4D), a result consistent with the fact that *laminin1* is expressed by cells surrounding the eye field (Figures S4E and S4F).

Laminin1 is not only required but is also sufficient to instruct the polarization of the eye field because cells oriented their apical surfaces away from Laminin1-coated beads implanted in the core of the eye field (n = 24, Figures 6H–6J and S4H; Movie S9). In contrast, BSA-coated beads at the center of the eye field had no effect on the polarization of eye field cells (n = 12; Figures 6K–6M and S4G; Movie S9). Thus, Laminin1 has an essential role in promoting and maintaining the organization of the marginal eye field cells as a pseudostratified neuroepithelium.

### *pard6γb* and *laminin-γ1* Synergize to Promote Optic Vesicle Evagination

Despite defects in the organization of the eye field cells as a pseudostratified neuroepithelium in the absence of Laminin1, optic vesicles still evaginate (albeit with some delay, Figures 6B, S4I, and S4J), illustrating the robustness of this morphogenetic process. Polarization does still occur in the absence of Laminin1, and so to assess if this is required for evagination, we disrupted AB polarization through reducing activity of Pard6γb using morpholino oligonucleotides (Munson et al., 2008). Cell polarization is indeed compromised in *pard6γb* morphants, as evident by the absence of ZO-1 staining in some eye field cells (Figure 6Nii). As in *laminin-γ1* mutants/morphants, optic vesicles in *pard6γb* morphants still evaginate with some delay (Figure S4K). However, simultaneous abrogation of *laminin-γ1* and *pard6γb* had a much more severe effect on morphogenesis and resulted in a failure of optic vesicle evagination and cyclopia by 24 hpf (Figures 6Niii and S4L). The cyclopic phenotype is unlikely to be a consequence of cell death because the phenotype was still present when p53-dependent apoptosis was blocked. These results show that the concomitant abrogation of genes involved in two distinct aspects of epithelialization leads to a failure in evagination of the optic vesicles and support the conclusion that eye field cells must apicobasally polarize and coordinate their polarity for optic vesicle evagination to occur.

## Discussion

In this study, we show that the eye field comprises two populations of cells that show distinct morphologies, organization, and behaviors and that contribute to different aspects of optic vesicle morphogenesis. Basally positioned cells establish a pseudostratified neuroepithelium at the margins of the evaginating eye field well before such organization is apparent in adjacent neural tissue. The eye field consequently shows precocious neuroepithelial organization as compared to the rest of the CNS, and this is associated with a morphogenetic behavior distinct from other regions. The second population of cells is located at the core of the eye field and is mesenchymal in morphology during the period when the basal cells epithelialize. Core cells establish apical anchor points and undergo MET during which they elongate and intercalate between cells of the basal, epithelialized domain of the optic vesicle thereby contributing to the later steps of optic vesicle evagination. Once the eye field cells have incorporated into the evaginating optic vesicles, shortening and reorientation of optic vesicle cells continue shaping the forming eyes.

**Figure 7:**
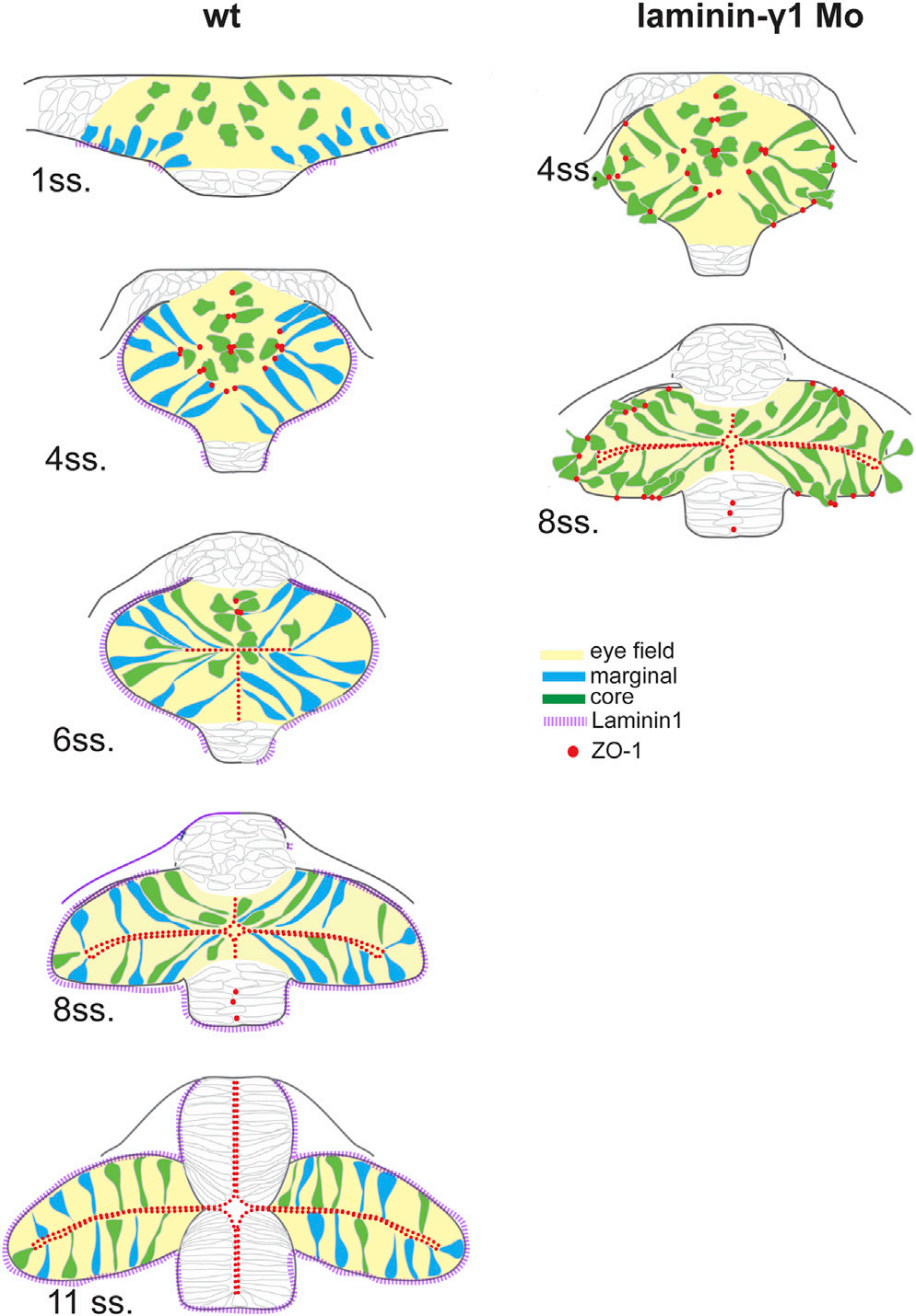
A Model for Cell Reorganization during Optic Vesicle Evagination Eye field cellular organization during morphogenesis in a wild-type (left column) and *lamininγ1* morphant embryo (right column). In the wild-type eye field, cells organize and polarize precociously as compared to cells in other regions of the neural plate. Cells at the margin of the eye field (blue) acquire a monolayered epithelium-like organization from 4 ss, prior to the onset of optic vesicle evagination; subsequently, cells at the core of the eye field (green) integrate into the marginal epithelium by intercalating among the marginal cells. At late stages of optic vesicle morphogenesis, eye cells shorten and acquire the final dorsoventral orientation characteristic of optic vesicle neuroepithelial cells. In *laminin-γ1* morphants, a subset of eye field cells fails to organize within the neuroepithelium, showing uncoordinated AB cell polarity.

Our analyses of the early steps in eye morphogenesis (summarized in Figure 7) add to previous observations (Rembold et al., 2006) that showed eye field cells elongating and apparently migrating as individuals during optic vesicle evagination rather than undergoing epithelial remodeling. One possibility is that the actively migrating cells imaged by Rembold et al. (2006) may be equivalent to the core cells that we describe here, and rather than undergoing individual migration, it is the process of intercalation that incorporates these cells into the forming optic vesicles. Some active cell migration may still happen during eye morphogenesis because, for instance, eye field cells initially inappropriately positioned within the prospective telencephalon appear to move into the forming eyes (Cavodeassi et al., 2013). The observation that evagination of the optic vesicles is compromised by concomitant interference with the establishment and coordination of AB polarity (by abrogating both Pard6γb and Laminin1 function) further supports the notion that epithelial organization is required for evagination. Therefore, epithelial remodeling is likely to be a common feature of optic vesicle evagination throughout vertebrates. Although not analyzed in great detail, it is likely that neural plate cells organize as a neuroepithelium prior to the onset of eye morphogenesis in mouse (Svoboda and O’Shea, 1987), whereas in zebrafish, only a subset of eye field cells acquires a neuroepithelial organization at the onset of eye field morphogenesis. The remaining eye cells epithelialize as they intercalate into the forming optic vesicles (this study; Sinn and Wittbrodt, 2013). It will be interesting to study comparable events in early eye formation in *Xenopus* because similar radial intercalation of deep cells into the more superficial neural epithelium has been observed within the spinal cord (Edlund et al., 2013). The steps required for optic vesicle evagination (epithelialization, cell shape changes, intercalation) can probably vary in relative timing between species, but the cell biology of each event is likely conserved. In vivo analyses of cell dynamics will need to be performed in other model systems to fully resolve the similarities and differences in optic vesicle evagination between zebrafish and other species.

Marginal eye field cells precociously acquire a cellular organization typical of a pseudostratified neuroepithelium by the coordination of several morphogenetic processes. First, individual marginal cells elongate and coordinate their cellular lengths with those of their neighbors to establish a cellular monolayer. Second, they acquire AB polarity along a common axis, as evidenced by the apical localization of polarity proteins, tight and adherens (data not shown) junctional components, and centrosomes. Third, they adopt spindle cell shapes, a characteristic of apicobasally pseudostratified neuroepithelial cells. Fourth, marginal cells start to undergo mitosis apically with both daughter cells subsequently elongating and reintegrating among their neighbors, a behavior typical of neuroepithelial cells undergoing IKNM (Lee and Norden, 2013).

Precocious acquisition of AB polarity in the eye field is likely to be a downstream consequence of the action of a network of transcription factors that specify the eye field (Agathocleous and Harris, 2009). Indeed, transcription of *pard6γb* occurs earlier in the eye field than the rest of the neural plate, and this early onset of expression is lost in the absence of the eye field transcription factor Rx3 (Figure S1F). These observations suggest that the eye field transcription factor network may override the intrinsic developmental timer that is proposed to regulate the schedule of epithelial polarization elsewhere in the neural plate (Girdler et al., 2013). Eye field transcription factors and other genes (such as *pard6γb*) are expressed throughout the eye field, suggesting a homogeneity among both prospective marginal and core eye field cells. We suggest that the distinction between these two cell populations is instead due to their apposition to, or separation from, the Laminin1-enriched basal lamina.

Laminin1 is not required for marginal cells to acquire AB polarity, but it can orient AB polarity and is consequently required for coordinated AB organization of the eye field neuroepithelium. Laminin1 shows strong accumulation around the eye field coincident with the epithelial organization of the marginal cells, and abrogation of Laminin1 results in many marginal eye field cells adopting aberrant morphologies and misoriented AB polarity axes. These aberrant morphologies seem to be the result of the inability of eye field cells to maintain rather than to establish an elongated shape in the absence of Laminin1. It is therefore likely that the elongation process per se is not solely under the control of Laminin1 and that the elongation and organization of neuroepithelial cells may be uncoupled from each other. These observations are consistent with a role for Laminin1 in facilitating the normal progression of organogenesis through coordinating AB polarity as suggested in other contexts (Buckley et al., 2013; Grant and Moens, 2010; Huang et al., 2003; O’Brien et al., 2001; Rasmussen et al., 2012; Yarnitzky and Volk, 1995). Indeed, at the level of the zebrafish hindbrain, neural cells assemble an apical domain at the nascent midline, as distant as possible from the Laminin1-containing basal lamina (Buckley et al., 2013).

In the absence of Laminin1, many eye field cells still establish epithelial domains with coherent AB organization, and this level of organization is presumably sufficient to support the compromised and delayed optic vesicle evagination that still occurs in Laminin mutants (Biehlmaier et al., 2007; Lee and Gross, 2007; Zolessi et al., 2006). Similarly, abrogation of the polarity protein Pard6γb alone does not fully compromise AB polarization, and optic vesicle formation. Concomitant disruption of genes (*pard6γb* and *laminin-γ1*) that contribute to different aspects of epithelialization in the eye field does, in contrast, lead to a failure of optic vesicle evagination. Similarly, multiple mechanisms have also been proposed to contribute to the formation of the neural tube epithelium in fish. In addition to receiving polarization cues from the basal lamina, neural cells may possess an intrinsic timer and have also the ability to sense the nascent tissue midline through cell-to-cell contacts (Buckley et al., 2013; Girdler et al., 2013) that, when coupled with c-divisions, may help to ensure error-free epithelialization of the neural keel.

Despite not being in contact with the basal lamina, cells located at the core of the eye field display coordinated behaviors. They localize Pard3 in a polarized manner and establish apical-to-apical contact points with neighboring core and marginal cells. Core cells maintain apical contact points as they undergo intercalation, suggesting that such contacts may anchor the cells to ensure that they insert into the nascent epithelium of the evaginating optic vesicles with the correct axis of AB polarity.

The intercalation of core eye field cells into the nascent epithelium of the evaginating optic vesicles constitutes an unusual MET. Other well-studied METs such as formation of the renal and gut epithelium (Dush and Nascone-Yoder, 2013; Urban et al., 2006; Vize et al., 1997) and somite formation from the presomitic mesoderm (Barrios et al., 2003; Watanabe et al., 2009) involve the establishment of an epithelium from a homogeneous population of mesenchymal cells. Perhaps comparable to the scenario we describe in the eye is the mechanism proposed to underlie gut formation in frogs (Dush and Nascone-Yoder, 2013) or lumen formation during secondary neurulation in chick, where it is thought that the cells in the center of the cord intercalate in between the already epithelialized cells of the outer neural layer (Schoenwolf and Delongo, 1980). Despite the apparent similarities with the process we describe, however, secondary neurulation has not been studied with sufficient detail to take the comparison further. For example, it is unclear whether central cells in the spinal cord are polarized prior to the intercalation step as is the case for core eye field cells.

Core eye field cells intercalate gradually, starting at the onset of optic vesicle evagination and continuing over a prolonged period of time. Early fate map studies in *Xenopu*s already suggested extensive cell movements during optic vesicle formation (Eagleson et al., 1995; Jacobson and Hirose, 1978). For instance, cells that originate from the same region of the neural plate could disperse throughout the mature optic cup and could occasionally cross the midline. In addition, movement of cells into the prospective retina during late stages of optic vesicle/cup formation is documented in both frogs and fish (Holt, 1980; Jacobson and Hirose, 1978; Kwan et al., 2012; Picker et al., 2009). These studies have shown that there is a “flow” of cells from the optic stalk into the ventral retina and around the rim of the optic cup into the temporal retina. Complex cellular movements and rearrangements are consequently likely to be a general feature of optic vesicle formation in vertebrates. Intercalating core cells are unlikely to be predetermined to populate any particular region within the forming eyes: cells intercalating at the onset of optic vesicle evagination will most likely occupy regions of the marginal layer that will later be located distally in the optic cup, whereas cells intercalating later are more likely to colonize medial regions of the optic vesicle such as the optic stalk. Consequently, once evagination is complete, core cells are distributed throughout the optic vesicles and are likely to contribute to all the domains of the mature eye.

Previous analyses suggest that regionalization of the optic vesicles is mediated by secreted signals that act on cells during or subsequent to their evagination (Ekker et al., 1995; Kruse-Bend et al., 2012; Lupo et al., 2005; Macdonald et al., 1995; Martinez-Morales and Wittbrodt, 2009; Picker and Brand, 2005; Picker et al., 2009; Take-uchi et al., 2003; Veien et al., 2008). For example, studies in zebrafish have shown that the regionalization into nasal and temporal retinal domains is mediated by Fgf signals secreted by dorsal neural and nonneural tissues, and this regional distinction is only established once the evagination process is well underway (Picker and Brand, 2005; Picker et al., 2009). This relatively late allocation of regional identity makes sense given the extensive cell movements that radically alter cell positions during morphogenesis of the eyes.

Recent studies have reported the formation of adenohypophyses, thyroid follicle, optic vesicles, and cerebral organoids in vitro using 3D cultures of ESC aggregates (Antonica et al., 2012; Eiraku et al., 2011; Lancaster et al., 2013; Suga et al., 2011). Whereas the formation of a functional thyroid follicle or adenohypophysis in vitro required the interaction of more than one tissue, optic vesicles self-organize spontaneously from homogeneous aggregates of pluripotent cells when cultured in a Laminin-rich Matrigel. Although the cellular behaviors that accompany optic vesicle formation from ESCs have not been analyzed in detail, they must involve many of the same general processes that we describe here. Indeed, the successful formation of optic vesicles from ESCs is dependent upon a Laminin-rich Matrigel supporting the idea that this matrix protein provides an essential scaffold upon which eye field cells can organize during the initial steps of optic vesicle formation. Consequently, we expect that our analyses of eye field cell behaviors will shed light not only upon the mechanisms of eye formation in vivo but also upon the remarkable processes by which ESCs can be coached to epithelialize, form optic vesicles, and differentiate as retinal neurons.

## Experimental Procedures

### Zebrafish

*AB* and *tupl* wild-type zebrafish strains, transgenic lines *Tg(rx3:GFP)*^*ET95/1*^ (Brown et al., 2010), *Tg(emx3:YFP)*^*b1200*^ (Viktorin et al., 2009), and *Tg(β-actin:HRAS-EGFP)* (Cooper et al., 2005), and the mutant strains *sleepy* (*sly*^*m86*^; Parsons et al., 2002) and *rx3/chk*^*ne2611*^ (Stigloher et al., 2006) were maintained and bred according to standard procedures (Westerfield, 1993). All experiments conform to the guidelines from the European Community Directive and the British legislation for the experimental use of animals.

### Immunolabeling and mRNA Detection

Whole-mount immunolabeling was performed as previously described (Wilson et al., 1990; see Supplemental Experimental Procedures for detailed antibody information). Membranes were highlighted by incubation with phalloidin coupled to 488/568 fluorophores (Molecular Probes), and nuclei were labeled with DAPI (Molecular Probes).

Antisense mRNA probes for whole-mount in situ hybridization were synthesized with T3 RNA polymerase (Promega) and digoxigenin-labeled nucleotides (Roche), following manufacturer’s instructions. Fluorescent whole-mount in situ hybridization was performed as previously described by Jülich et al. (2005). For detection, the embryos were incubated with anti-digoxigenin-POD (Roche) and developed using Cy3-TSA (PerkinElmer) as a substrate.

### Microinjection, Cell Transplantation, and Bead Implantation

Embryos were microinjected at one-cell stage with the required mRNA/morpholino (see Supplemental Experimental Procedures); in some cases (described in the figure legends), the injections were performed at later stages to generate mosaics. Cell transplantation was performed as described (Cavodeassi et al., 2005). Both donor and host embryos were at mid-to-late blastula stage at the time of the procedure. Six of 15 μm polystyrene beads (Invitrogen) were prepared as previously described by Randlett et al. (2011) and Regan et al. (2009) and implanted in early/midgastrula embryos using the same setup as for cell transplantation. Manipulated embryos were left to develop until the required stage and were then either fixed and prepared for immunostaining/in situ hybridization or mounted for live imaging and analysis.

### Imaging and Data Processing

Live and fixed embryos were embedded in low melting point agarose at 1% in PBS or embryo medium for frontal confocal imaging using a 40× (0.8 NA) long-working distance water-immersion lens, for imaging using a SPE Leica confocal microscopy system (see detailed description in Supplemental Experimental Procedures). Embryos subjected to in situ hybridization were additionally mounted flat in a drop of glycerol, and dorsal images were acquired with a 40× (1.3 NA) oil-immersion lens in a Zeiss LSM710 confocal microscope. Raw data were processed and analyzed with Volocity software (Improvision; see Supplemental Experimental Procedures).

## Author Contributions

K.I. performed the experiments. All authors contributed to the design of the study, interpretation of data, and writing of the manuscript.

## Supplemental Information

Document S1. Supplemental Experimental Procedures and Figures S1–S4

Movie S1. Eye Field Cells Only Rarely Cross the Midline, Related to Figure 1 Time-lapse of a single confocal plane of an embryo expressing Kaede in which half of the ANP was photoconverted from green to red. Some rare examples of crossing eye cells are highlighted throughout the movie (arrows). Crossing cells are more commonly observed in the hypothalamic and telencephalic territories (arrows). Interval between frames: 7 min 12 s.

Movie S2. Changes in Cellular Shape during Optic Vesicle Evagination, Related to Figure 2 Time-lapse of a single confocal plane of an embryo expressing Kaede in which half of the ANP was photoconverted from green to red. Some rare examples of crossing eye cells are highlighted throughout the movie (arrows). Crossing cells are more commonly observed in the hypothalamic and telencephalic territories (arrows). Interval between frames: 7 min 12 s.

Movie S3. Establishment of the Apical Domain in Marginal Cells, Related to Figure 3 Time-lapse of an embryo mosaically expressing pard3-GFP with all the membranes labeled by RFP. Z projection of five sections covering 11 μm. Interval between frames: 4 min 20 s.

Movie S4. Polarized Accumulation of pard3-GFP by a Core Cell, Related to Figure 4 Time-lapse of an embryo mosaically expressing pard3-GFP with all the membranes labeled by RFP. Note the polarized accumulation of pard3-GFP by the labeled core cell (yellow arrow). Z projection of six sections covering 20 μm. Interval between frames: 8 min 15 s.

Movie S5. Rosette Organization of Core Eye Field Cells, Related to Figure 4 Time-lapse of an embryo mosaically expressing pard3-GFP with all the membranes labeled by RFP. Note the coordinated convergence of the pard3-GFP puncta into a single punctum as the cells organize as a rosette (arrows). Z projection of four sections covering 14.67 μm. Interval between frames: 4 min 20 s.

Movie S6. Apical and Core Cells Stabilizing an Apical Contact Point, Related to Figure 4 Time-lapse of an embryo mosaically expressing pard3-GFP with all the membranes labeled by RFP. Note the maintenance of an apical contact point (yellow arrow) between the marginal and core cells (white arrows). Z projection of six sections covering 13.20 μm. Interval between frames: 4 min 20 s.

Movie S7. Core Cell Intercalation during Optic Vesicle Evagination, Related to Figure 4 Time-lapse of an embryo expressing Kaede in which only core cells have been photoconverted from green to red. Core cells gradually integrate in the evaginating optic vesicles by intercalation. Some example cells are followed throughout the movie (arrows). Z projection of two sections covering 10 μm. Interval between frames: 6 min 37 s.

Movie S8. Apical Anchoring of Intercalating Core Cells, Related to Figure 4 Time-lapse of an embryo mosaically expressing pard3-GFP with all the membranes labeled by RFP. Note the anchoring of the apical domain of the core cell (arrow) as it extends basally and intercalates. Z projection of three sections covering 9 μm. Interval between frames: 2 min 25 s.

Movie S9. Laminin1-Coated Beads Implanted in the Eye Field Promote Cell Polarity Reorganization, whereas BSA-Coated Beads Do Not, Related to Figure 6 Left: Time-lapse of an embryo in which Laminin1-coated beads (blue patch) have been implanted in the center of the eye field. Eye cells around the beads organize with their apical domains oriented away from the beads. The final time point of the movie is shown at different z levels to fully illustrate the reorganization of the cells. Right: Time-lapse of an embryo in which BSA-coated beads (blue patch) were implanted in the center of the eye field. Unlike Lamin1-coated beads, cells do not reorient their apicobasal polarity in response to the BSA beads. Both movies show a single confocal z section. Interval between frames: 6 min.
