# Supplementary material for: Precocious Acquisition of Neuroepithelial Character in the Eye Field Underlies the Onset of Eye Morphogenesis
Source: Dev Cell. 2013 Nov 11;27(3):293–305. doi: 10.1016/j.devcel.2013.09.023 (PMC3898423; doi:10.1016/j.devcel.2013.09.023)
Supplement: Document S1. Supplemental Experimental Procedures and Figures S1–S4 [file mmc1.pdf]

Developmental Cell, Volume *27*

Supplemental Information

Precocious Acquisition of Neuroepithelial Character in the Eye Field Underlies the Onset of Eye Morphogenesis

Kenzo Ivanovitch, Florencia Cavodeassi, and Stephen W. Wilson

Supplemental information inventory

Supplemental figures S1-S4

- **Figure S1, related to Figure 1:** Apical markers in the eye field.
- **Figure S2, related to Figure 3:** Establishment of apicobasal polarity in the telencephalon.
- **Figure S3, related to Figure 4:** pard3-GFP dynamics in core cells.
- **Figure S4, related to Figure 6:** lack of Laminin compromises optic vesicle evagination.

Supplemental movies legends

- **Movie S1, related to Figure 1.** Eye field cells only rarely cross the midline.
- **Movie S2, related to Figure 2.** Changes in cellular shape during optic vesicle evagination.
- **Movie S3, related to Figure 3.** Establishment of the apical domain in marginal cells.
- **Movie S4, related to Figure 4.** Polarised accumulation of pard3-GFP by a core cell.
- **Movie S5, related to Figure 4.** Rosette organisation of core eye field cells.
- **Movie S6, related to Figure 4.** Apical and core cells stabilising an apical contact point.
- **Movie S7 related to Figure 4.** Core cell intercalation during optic vesicle evagination.
- **Movie S8, related to Figure 4.** Apical anchoring of intercalating core cells.

- **Movie S9, related to Figure 6.** Laminin1-coated beads implanted in the eye field promote cell polarity reorganization, while BSA-coated beads do not.

Supplemental experimental procedures

Supplemental information:

Supplemental figures S1-S4

Figure S1, related to Figure 1: Apical markers in the eye field.

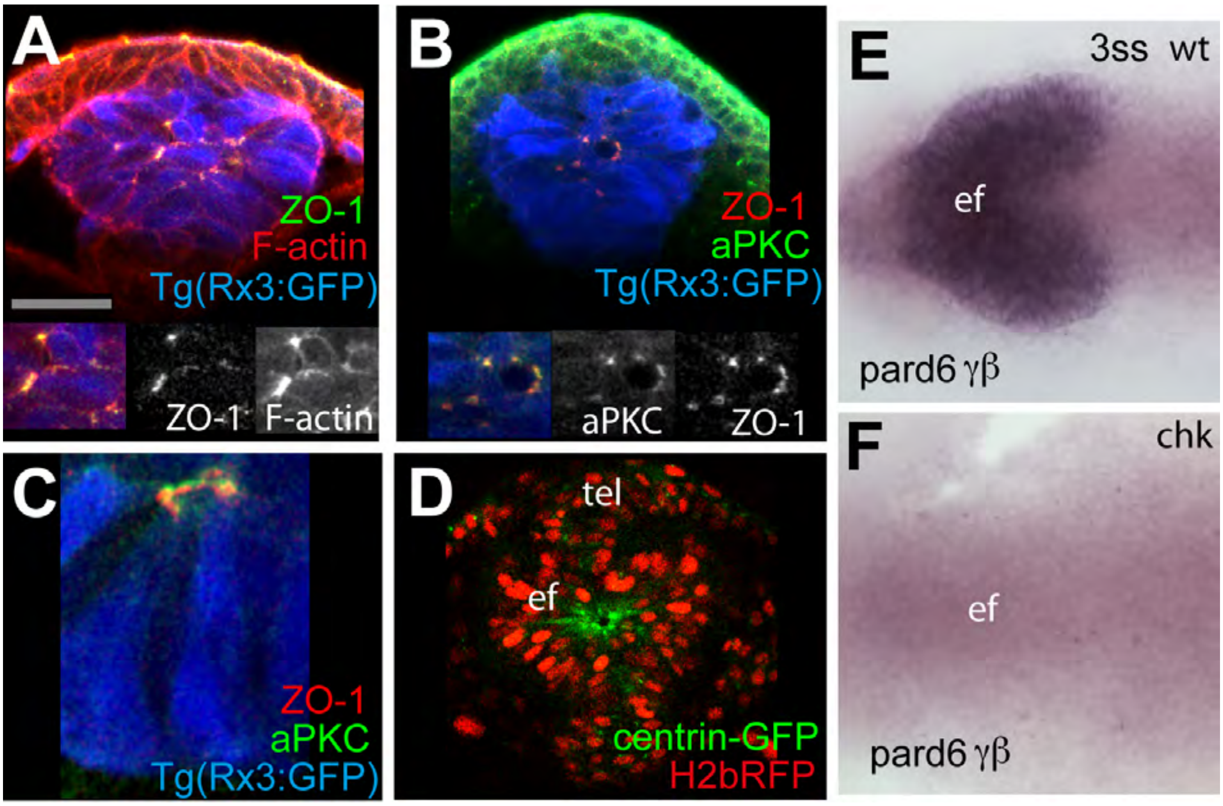

(A-C) 4ss Tg(rx3:GFP) embryos immunostained as detailed in the panels [ZO-1 (green in A, red in B and C), F-actin (red, A) and aPKC (green in B)]. Inset boxes showing F-actin and aPKC, and ZO-1 and aPKC colocalise in the eye field at 4ss. aPKC can sometimes localise apically to ZO-1 (C).

(D) View of the eyefield of a wildtype embryo injected with mRNA for H2b-RFP to label the nuclei and centrin-GFP to label centrosomes, showing accumulation of this marker at the centre of the eye field. White dotted lines in (D) outline the eye field. tel: telencephalon; ef: eye field.

(E-F) in situ hybridization to detect *pard6γβ* in the eye field of wildtype (E) and $rx3^{chk}$ mutant embryos (F).

Figure S2, related to Figure 3: Establishment of apicobasal polarity in the telencephalon.

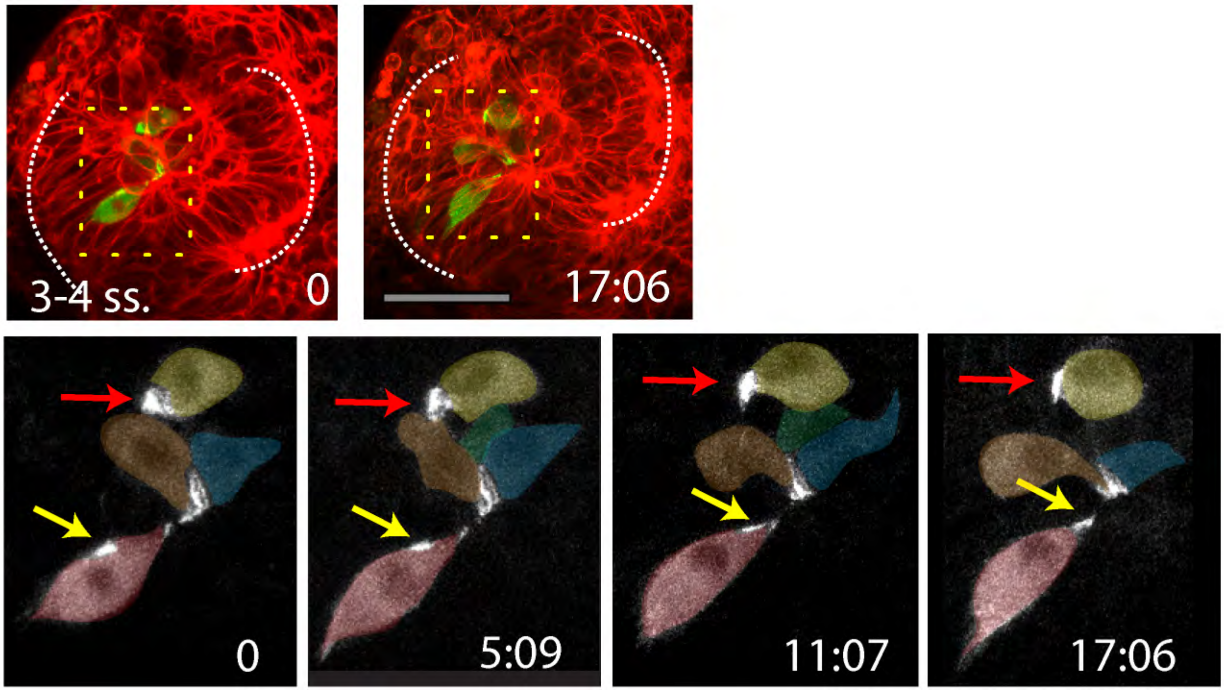

(A-B) 9ss (A) and 11ss (B) Tg(emx3:YFP) embryos immunostained as detailed in the panels. (Ai, ii, iii) and (Bi, ii, iii) show high magnifications of the telencephalic domain. ZO-1 is only weakly expressed at 9ss (Aii) but by 11ss, it is strongly expressed adjacent to the lumen of the telencephalon (Bii).

(C) Snapshots from a time lapse movie of a pard3-GFP mRNA injected embryo, at 9ss (i), 11ss (ii) and 12ss (iii), showing the gradual appearance of polarisation in the telencephalon.

(D) high-resolution detail of a telencephalic cell expressing lifeact-RFP and pard3-GFP, as it undergoes a midline cell division and its daughter cells establish apical domains at the telencephalic midline.

White dotted lines in (A-B) outline the eye field; lines in (Ai-iii; Bi-iii; Ci-iii) outline the telencephalon. Vertical dotted lines in (D) highlight the future midline.

Figure S3, related to Figure 4: pard3-GFP dynamics in core cells.

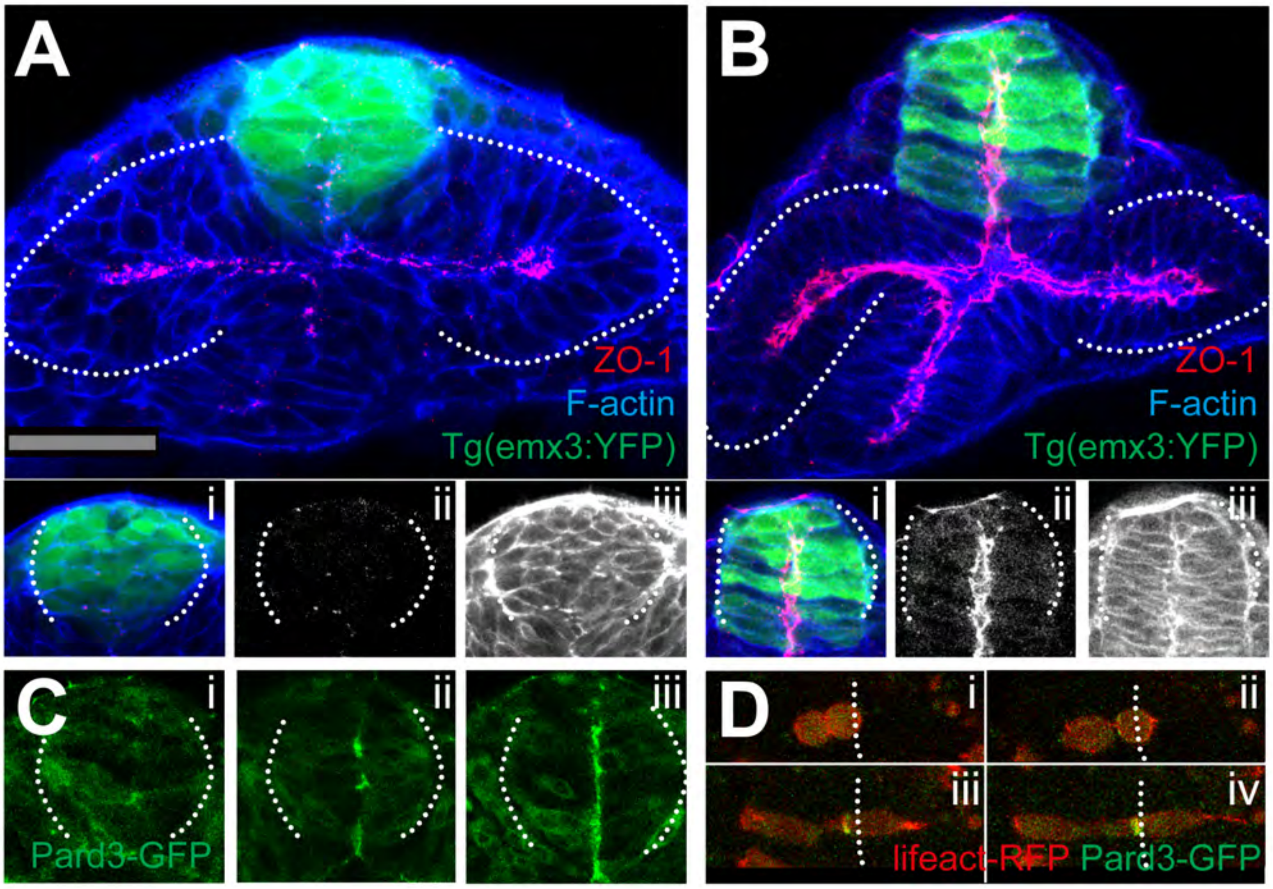

Time-lapse movie sequence of eye field core cells expressing the fusion protein Pard3-GFP (green). The embryo is counter-labeled with a membrane-RFP (red). Insets below show the green channel only (Pard3-GFP). A subset of the labelled core cells coordinate their subcellular polarised localisation of Pard3-GFP and organise as a rosette-like structure (follow the yellow arrow, n=5 in 4 embryos). Some others did not do so (red arrow).

Figure S4, related to Figure 6: lack of Laminin compromises optic vesicle evagination.

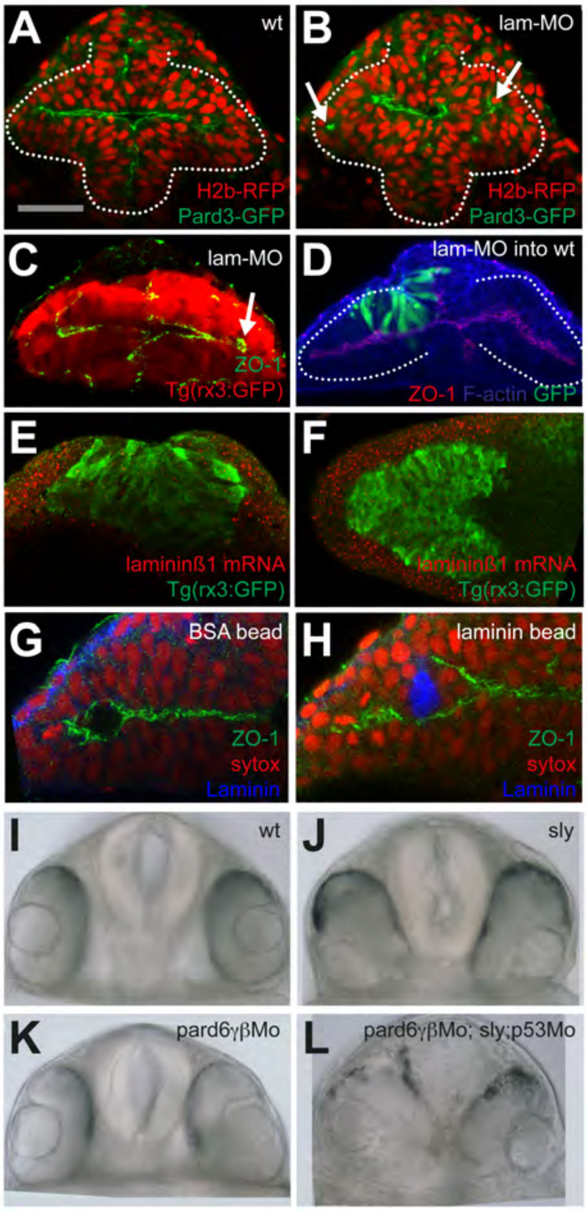

(A-B) wildtype and laminin-γ1 morphants, respectively, expressing H2b-RFP and Pard3-GFP. Arrows in (B) point at mis-positioned pard3-GFP puncta in the eye field of the laminin-γ1 morphant.

(C) laminin-γ1 morphant immunostained as detailed in the panel. Arrows point at mis-positioned ZO-1 puncta.

(D) laminin-γ1 morphant cells (green) transplanted into a wild type eye field show no overt phenotype. Embryo immunostained as detailed in the panel.

(E-F) frontal and dorsal views, respectively, of 3ss Tg(rx3:GFP) embryos showing *laminin-β1* mRNA accumulation (red) and GFP protein (green). *laminin-α1* and *laminin-γ1* show similar expression patterns (not shown).

(G-H) BSA-coated (G) and Laminin1-coated (H) beads implanted in the eye field. Embryos were left to develop until 10ss and immunostained as detailed in the panels.

(I-L) Frontal views of 24hpf embryos of the genotypes detailed in the panels.

Supplemental movies legends

Movie S1, related to Figure 1. Eye field cells only rarely cross the midline.
Time lapse of a single confocal plane of an embryo expressing Kaede, in which half of the ANP was photoconverted from green to red. Some rare examples of crossing eye cells are highlighted throughout the movie (arrows). Crossing cells are more commonly observed in the hypothalamic and telencephalic territories (arrows). Interval between frames: 7mn 12s.

Movie S2, related to Figure 2. Changes in cellular shape during optic vesicle evagination.
Time lapse of a single confocal plane of a Tg(β-actin:HRAS-EGFP) embryo where membranes are labelled with GFP and nuclei are labelled with RFP. Changes in cell shape and organisation can be followed as morphogenesis proceeds. Interval between frames: 4 mn 31s.

Movie S3, related to Figure 3. Establishment of the apical domain in marginal cells.
Time lapse of an embryo mosaically expressing pard3-GFP with all the membranes labelled by RFP. Z-projection of five sections covering 11µm. Interval between frames: 4 mn 20s.

Movie S4, related to Figure 4. Polarised accumulation of pard3-GFP by a core cell.
Time lapse of an embryo mosaically expressing pard3-GFP with all the membranes labelled by RFP. Note the polarised accumulation of pard3-GFP by the labelled core cell (yellow arrow). Z-projection of six sections covering 20µm. Interval between frames: 8 mn 15s.

Movie S5, related to Figure 4. Rosette organisation of core eye field cells.
Time lapse of an embryo mosaically expressing pard3-GFP with all the membranes labelled by RFP. Note the coordinated convergence of the pard3-GFP puncta into a single punctum as the cells organise as a rosette (arrows). Z-projection of four sections covering 14.67µm. Interval between frames: 4mn 20s.

Movie S6, related to Figure 4. Apical and core cells stabilising an apical contact point.
Time lapse of an embryo mosaically expressing pard3-GFP with all the membranes labelled by RFP. Note the maintenance of an apical contact point (yellow arrow) between the marginal and core cells (white arrows). Z-projection of six sections covering 13.20µm. Interval between frames: 4mn 20s.

Movie S7 related to Figure 4. Core cell intercalation during optic vesicle evagination.

Time lapse of an embryo expressing Kaede in which only core cells have been photoconverted from green to red. Core cells gradually integrate in the evaginating optic vesicles by intercalation. Some example cells are followed throughout the movie (arrows). Z-projection of two sections covering 10μm. Interval between frames: 6mn 37s.

Movie S8, related to Figure 4. Apical anchoring of intercalating core cells.
Time lapse of an embryo mosaically expressing pard3-GFP with all the membranes labelled by RFP. Note the anchoring of the apical domain of the core cell (arrow) as it extends basally and intercalates. Z-projection of three sections covering 9µm. Interval between frames: 2mn 25s.

Movie S9, related to Figure 6. Laminin1-coated beads, and not BSA-coated beads, implanted in the eye field promote cell polarity reorganization.
Left: time lapse of an embryo in which Laminin1-coated beads (blue patch) have been implanted in the centre of the eye field. Eye cells around the beads organise with their apical domains oriented away from the beads. The final timepoint of the movie is shown at different Z-levels to fully illustrate the reorganization of the cells.
Right: time lapse of an embryo in which BSA-coated beads (blue patch) were implanted in the centre of the eye field. Unlike Lamin1-coated beads, cells do no reorient their apicobasal polarity in response to the BSA beads. Both movies show a single confocal z-section. Interval between frames: 6mn

Supplemental experimental procedures

Immunolabelling and mRNA detection

The following antibodies were used for immunolabelling: chicken anti-GFP (1:1000, Abcam); mouse anti-ZO-1 (1:400, Molecular Probes); rabbit anti-laminin1 (1:400, Sigma); mouse anti-bcatenin (1:400, Sigma); rabbit anti-aPKC (1:250 dilution, Santa Cruz, CA, USA); and secondary antibodies (Molecular Probes) coupled to 488, 543 or 633 fluorophores as required.

cDNA templates for laminin-a1, -b1 and -g1 probes were amplified from embryonic cDNA using the following primers:

laminin-α1: fwd-AAAAACCACACCGACGGGCCT, rev-AATTAACCCTCACTAAAGGGTAGCGTAGCGTAGCGTGTCTCCCAAACCGC

laminin-β1: fwd-TGCTGTTACAACTCGCAGCCC, rev-AATTAACCCTCACTAAAGGGGCCAAACATGTGCTCCCGACTCG

laminin-γ1: fwd-GCTACGTCTGCCTTACACTC, rev-TCCATTAACCCTCACTAAAGGGAAAGCAGCAGCAAGCAGCGTC.

Microinjection

To phenocopy *laminin-g1* and *pard6gb* mutants, embryos were injected at 1-cell stage with approximately 0.7 picomoles/embryo of laminin-*g*1 morpholino (5'-TGTGCCTTTTGCTATTGCGACCTC-3'; Parsons et al., 2002) and 0.6 picomoles/embryos of *pard6gb* morpholino (5′ACATTCAACTCACCTTGCTTTTCAC-3′; Munson et al., 2008). To label cellular and subcellular structures, mRNA encoding for the following fusion proteins were injected: H2B-RFP (100-150pg), H2B-GFP (100-150pg), Pard3-GFP (von Trotha et al., 2006), 50-75pg), Gap43-mRFP (100-150pg), GAP43-mGFP (100-150pg), Kaede (Ando et al., 2002, 100-150pg).

Imaging and data processing

Embryos for live imaging were imaged from a frontal point of view by confocal fluorescence microscopy using a Leica SPE system in a chamber heated at 28.5°C. z-stacks 20 to 80µm thick were taken every 2-7 minutes for approximately 5 hours. Embryos injected with *kaede* mRNA were kept in the dark until subjected to green to red photoconversion. Kaede photoconversion was done on live embryos mounted as described by scanning across the region of interest with a UV (405nm) laser for 10-15 times.

Raw data were processed and analysed with Volocity software (Improvision). Individual cells were tracked manually, and movies were assembled by manual correction of the drift resulting from the

growth of the embryo. Photoshop CS2 (Adobe) was used to adjust brightness/contrast, color balance and saturation in the selected snapshots. Cell shape measurements were done on single cells, imaged in mosaically labelled embryos, reconstructed in three dimensions, and analysed with Volocity software. All statistical comparisons as indicated in figure legends were performed using Prism 4 (Graph-Paf Softwas Inc., San Diego, CA).

Artwork

All the cartoons and schematics accompanying the data have been generated in Adobe Illustrator.

References

Ando, R., Hama, H., Yamamoto-Hino, M., Mizuno, H., and Miyawaki, A. (2002). An optical marker based on the UV-induced green-to-red photoconversion of a fluorescent protein. Proc Natl Acad Sci U S A *99*, 12651-12656.

Munson, C., Huisken, J., Bit-Avragim, N., Kuo, T., Dong, P.D., Ober, E.A., Verkade, H., Abdelilah-Seyfried, S., and Stainier, D.Y. (2008). Regulation of neurocoel morphogenesis by Pard6 gamma b. Dev Biol *324*, 41-54.

Parsons, M.J., Pollard, S.M., Saude, L., Feldman, B., Coutinho, P., Hirst, E.M., and Stemple, D.L. (2002). Zebrafish mutants identify an essential role for laminins in notochord formation. Development *129*, 3137-3146.

von Trotha, J.W., Campos-Ortega, J.A., and Reugels, A.M. (2006). Apical localization of ASIP/PAR-3:EGFP in zebrafish neuroepithelial cells involves the oligomerization domain CR1, the PDZ domains, and the C-terminal portion of the protein. Dev Dyn *235*, 967-977.
