# Supplementary figures and images for: Precocious Acquisition of Neuroepithelial Character in the Eye Field Underlies the Onset of Eye Morphogenesis
Source: Dev Cell. 2013 Nov 11;27(3):293–305. doi: 10.1016/j.devcel.2013.09.023 (PMC3898423; doi:10.1016/j.devcel.2013.09.023)

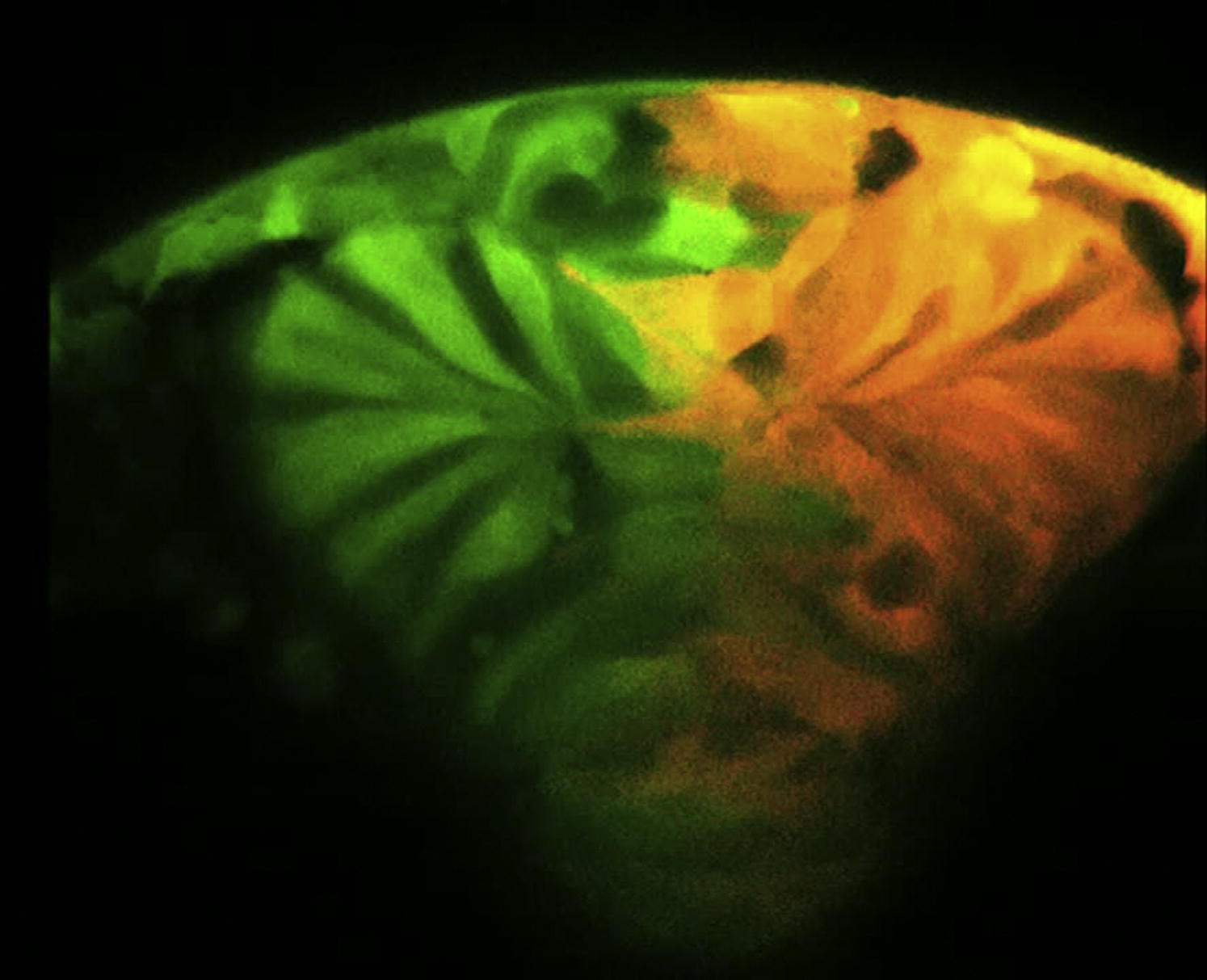

Supplement: Movie S1. Eye Field Cells Only Rarely Cross the Midline, Related to Figure 1 — Time-lapse of a single confocal plane of an embryo expressing Kaede in which half of the ANP was photoconverted from green to red. Some rare examples of crossing eye cells are highlighted throughout the movie (arrows). Crossing cells are more commonly observed in the hypothalamic and telencephalic territories (arrows). Interval between frames: 7 min 12 s. [file mmc2.jpg]

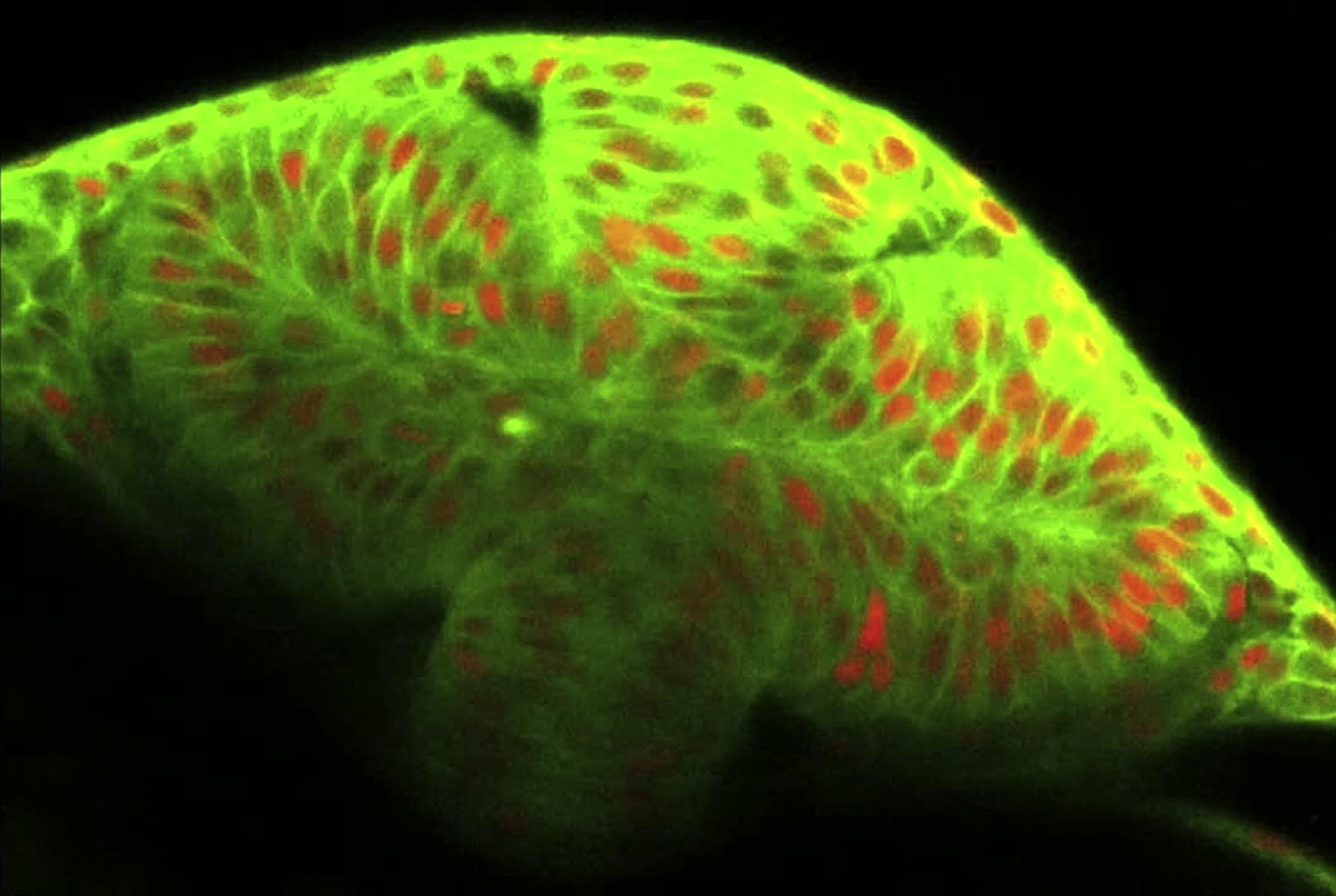

Supplement: Movie S2. Changes in Cellular Shape during Optic Vesicle Evagination, Related to Figure 2 — Time-lapse of a single confocal plane of an embryo expressing Kaede in which half of the ANP was photoconverted from green to red. Some rare examples of crossing eye cells are highlighted throughout the movie (arrows). Crossing cells are more commonly observed in the hypothalamic and telencephalic territories (arrows). Interval between frames: 7 min 12 s. [file mmc3.jpg]

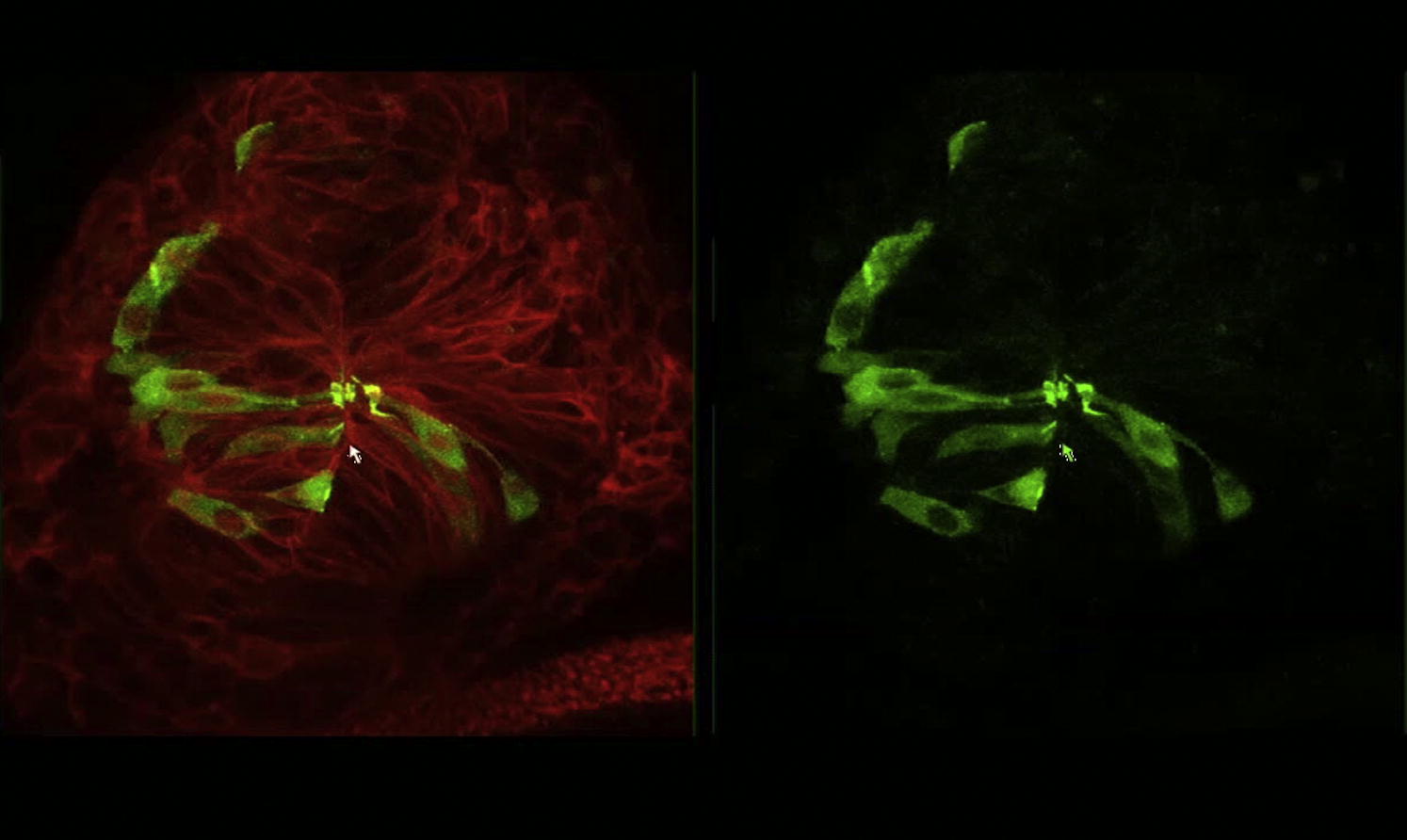

Supplement: Movie S3. Establishment of the Apical Domain in Marginal Cells, Related to Figure 3 — Time-lapse of an embryo mosaically expressing pard3-GFP with all the membranes labeled by RFP. Z projection of five sections covering 11 μm. Interval between frames: 4 min 20 s. [file mmc4.jpg]

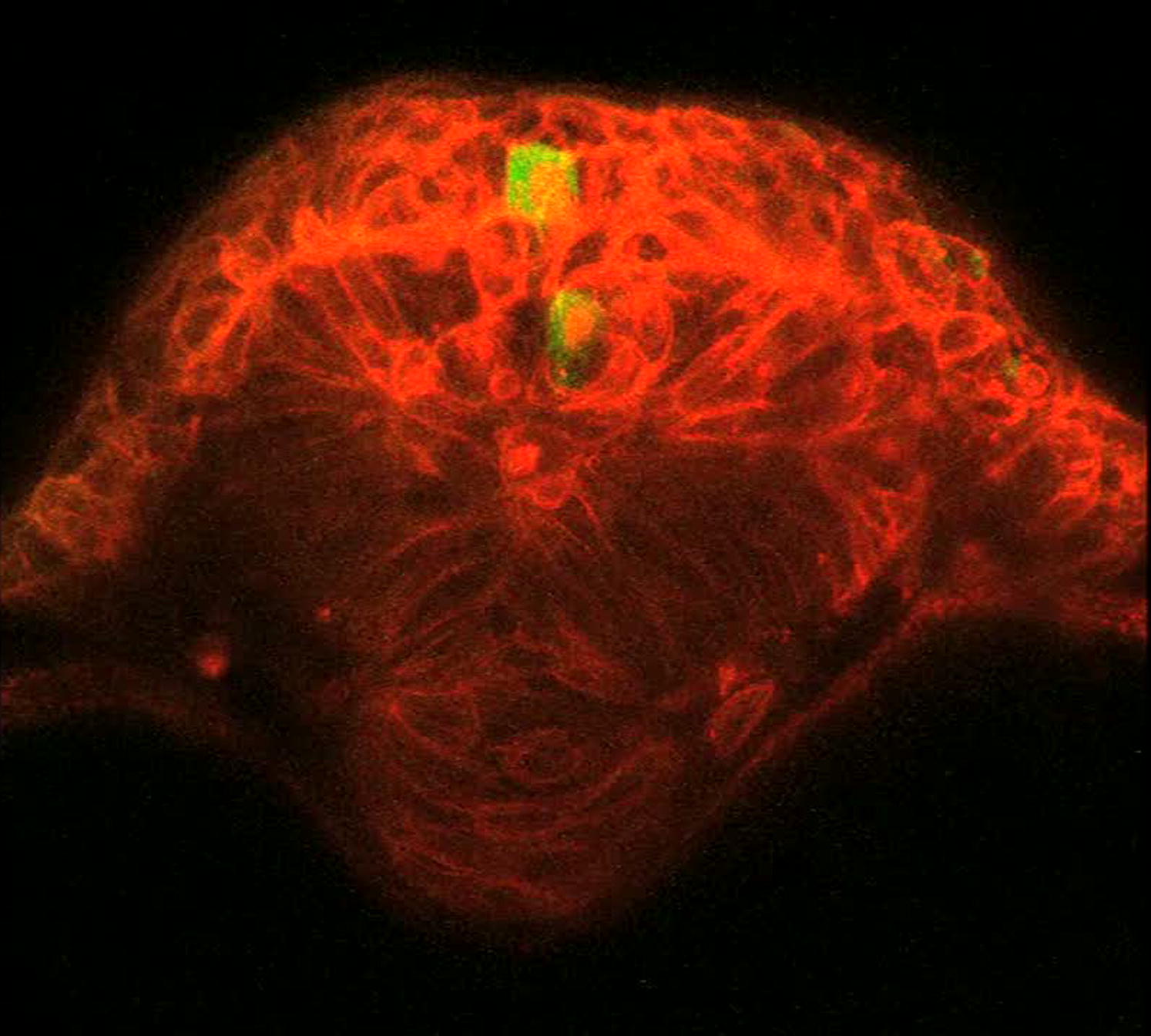

Supplement: Movie S4. Polarized Accumulation of pard3-GFP by a Core Cell, Related to Figure 4 — Time-lapse of an embryo mosaically expressing pard3-GFP with all the membranes labeled by RFP. Note the polarized accumulation of pard3-GFP by the labeled core cell (yellow arrow). Z projection of six sections covering 20 μm. Interval between frames: 8 min 15 s. [file mmc5.jpg]

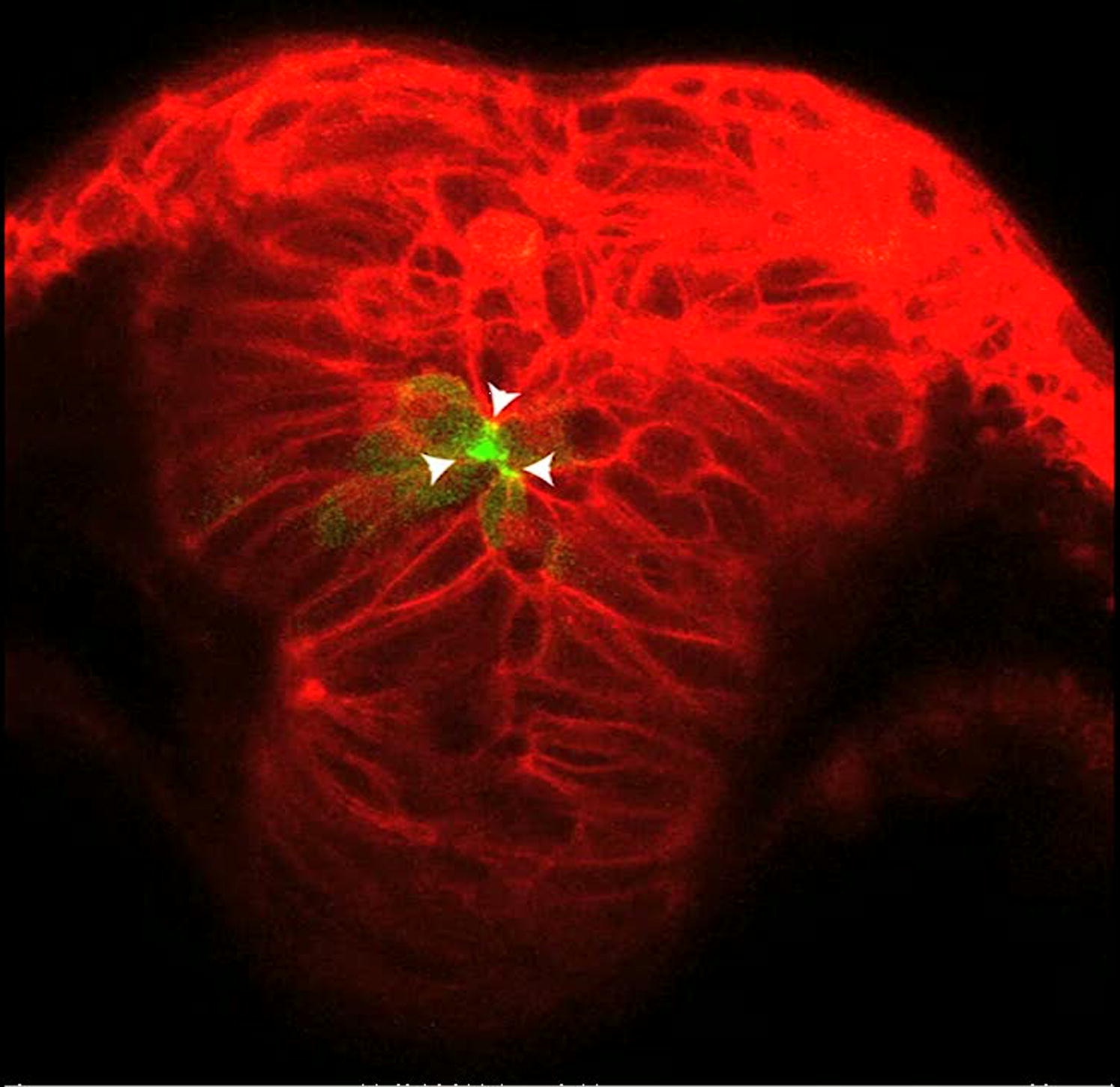

Supplement: Movie S5. Rosette Organization of Core Eye Field Cells, Related to Figure 4 — Time-lapse of an embryo mosaically expressing pard3-GFP with all the membranes labeled by RFP. Note the coordinated convergence of the pard3-GFP puncta into a single punctum as the cells organize as a rosette (arrows). Z projection of four sections covering 14.67 μm. Interval between frames: 4 min 20 s. [file mmc6.jpg]

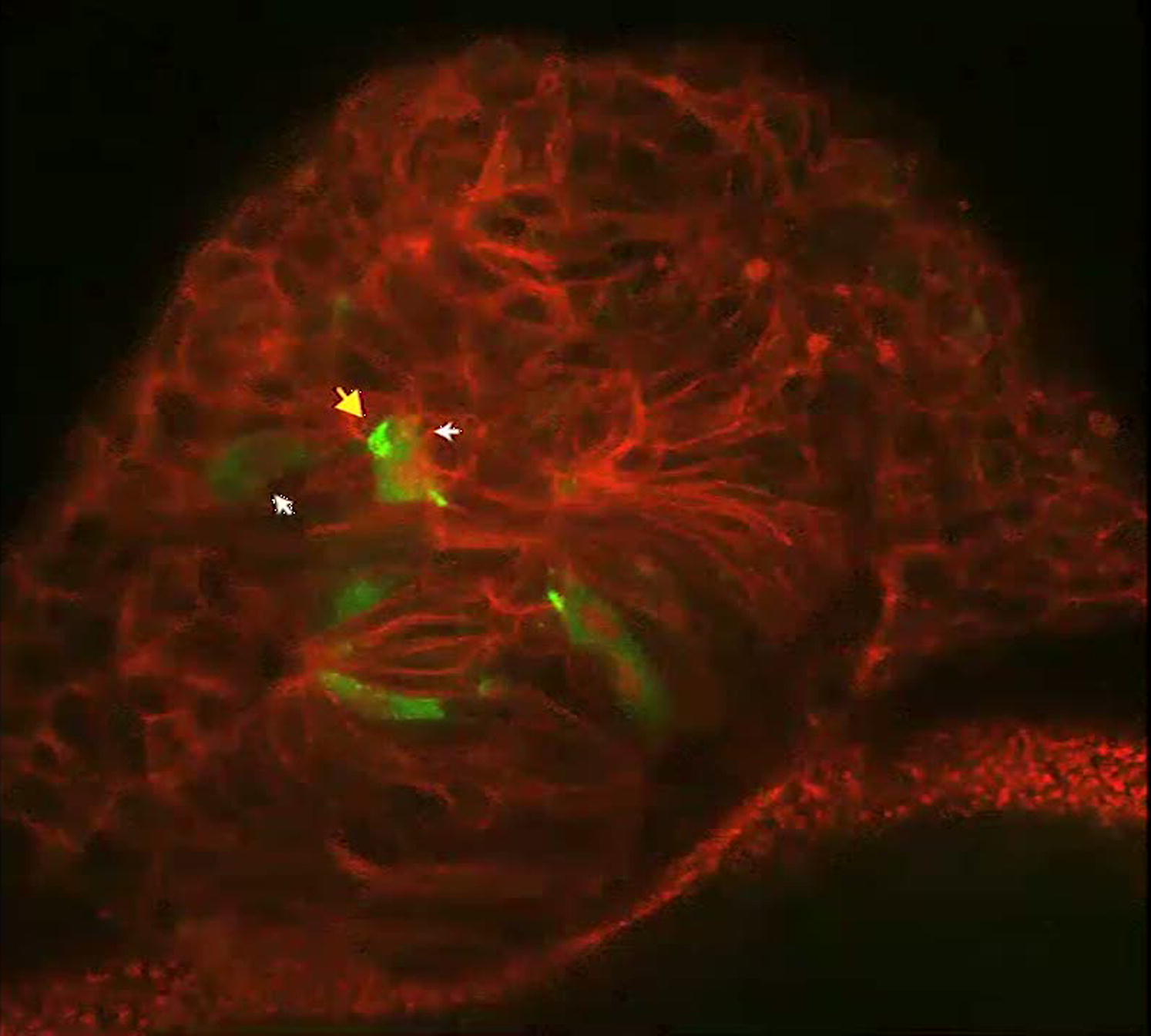

Supplement: Movie S6. Apical and Core Cells Stabilizing an Apical Contact Point, Related to Figure 4 — Time-lapse of an embryo mosaically expressing pard3-GFP with all the membranes labeled by RFP. Note the maintenance of an apical contact point (yellow arrow) between the marginal and core cells (white arrows). Z projection of six sections covering 13.20 μm. Interval between frames: 4 min 20 s. [file mmc7.jpg]

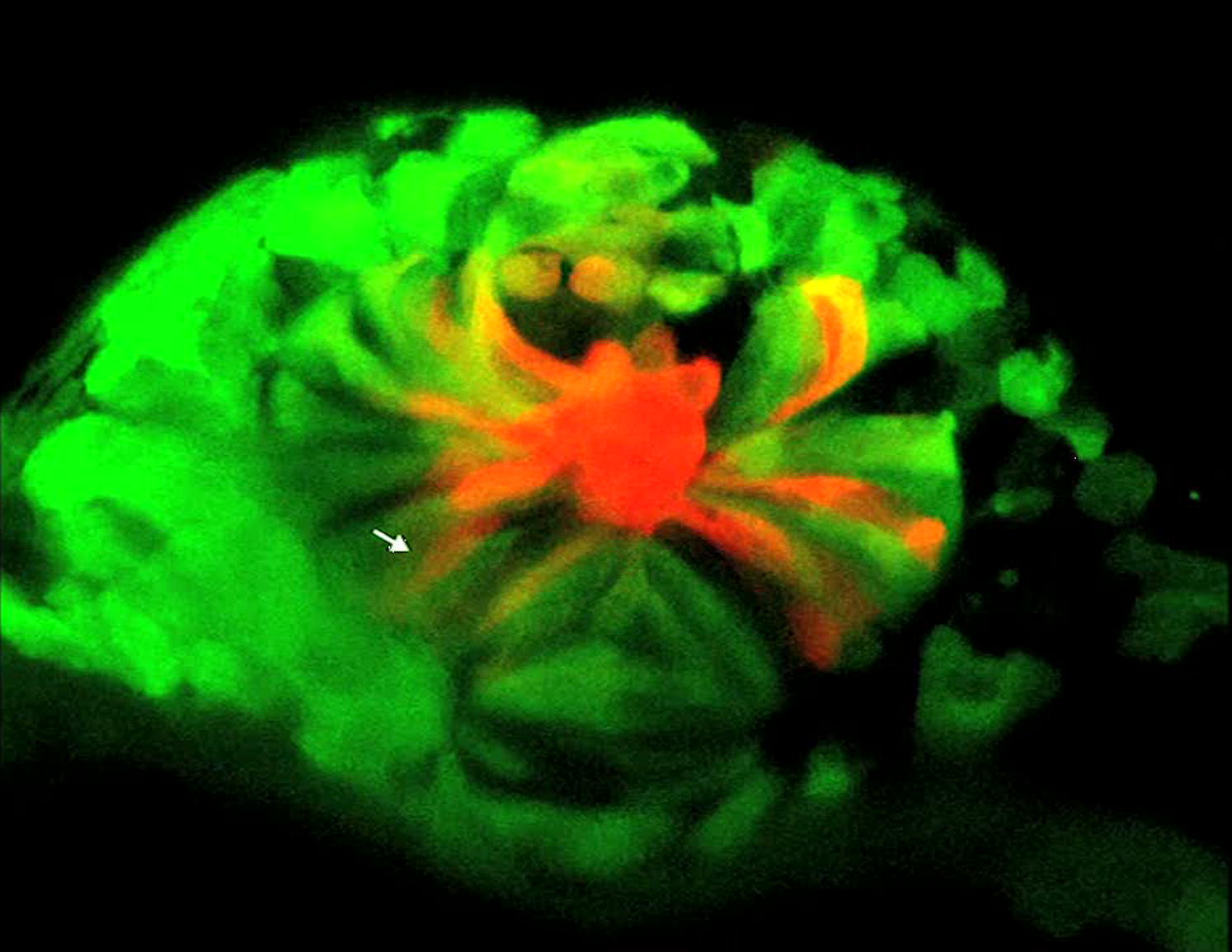

Supplement: Movie S7. Core Cell Intercalation during Optic Vesicle Evagination, Related to Figure 4 — Time-lapse of an embryo expressing Kaede in which only core cells have been photoconverted from green to red. Core cells gradually integrate in the evaginating optic vesicles by intercalation. Some example cells are followed throughout the movie (arrows). Z projection of two sections covering 10 μm. Interval between frames: 6 min 37 s. [file mmc8.jpg]

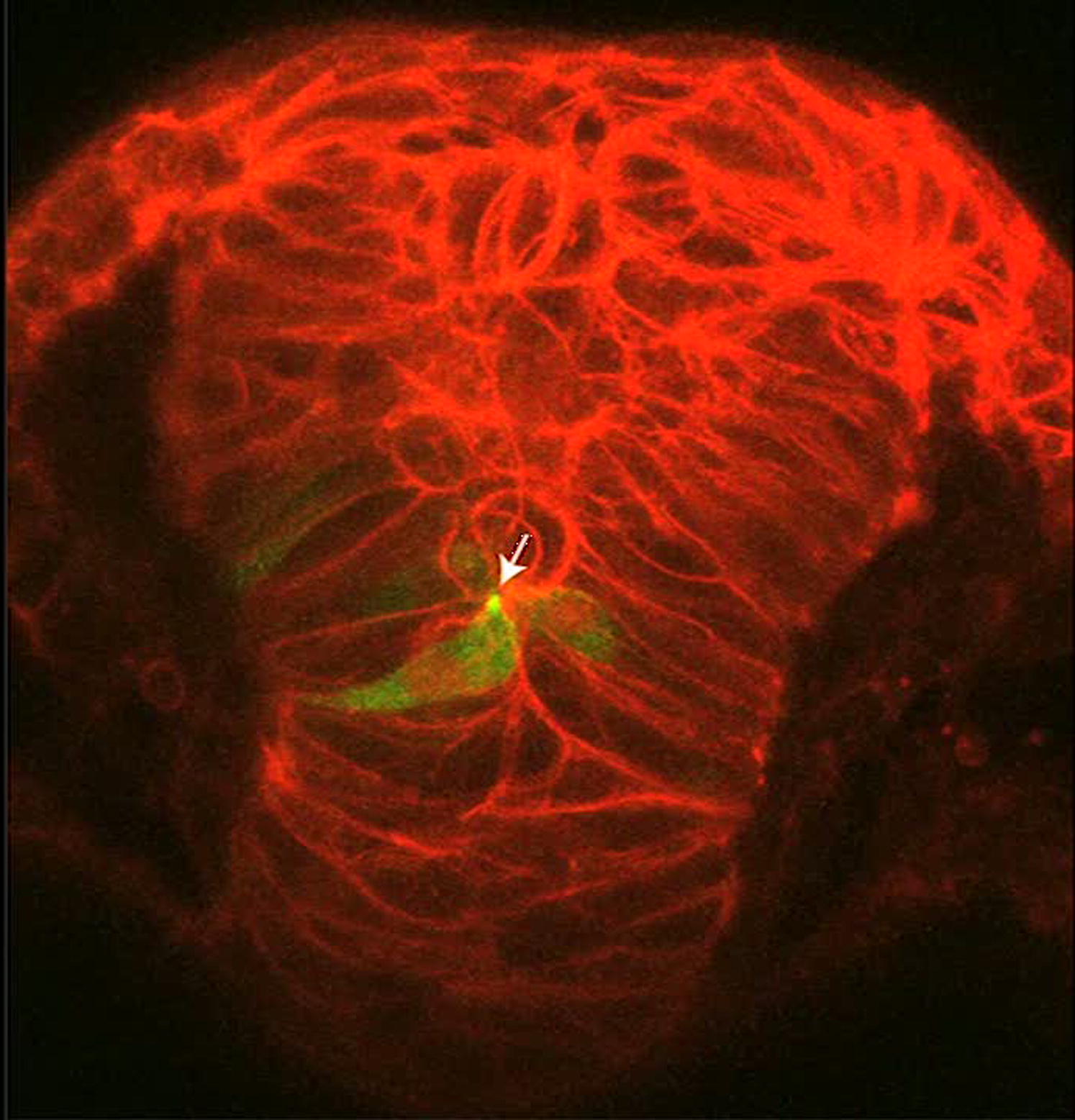

Supplement: Movie S8. Apical Anchoring of Intercalating Core Cells, Related to Figure 4 — Time-lapse of an embryo mosaically expressing pard3-GFP with all the membranes labeled by RFP. Note the anchoring of the apical domain of the core cell (arrow) as it extends basally and intercalates. Z projection of three sections covering 9 μm. Interval between frames: 2 min 25 s. [file mmc9.jpg]

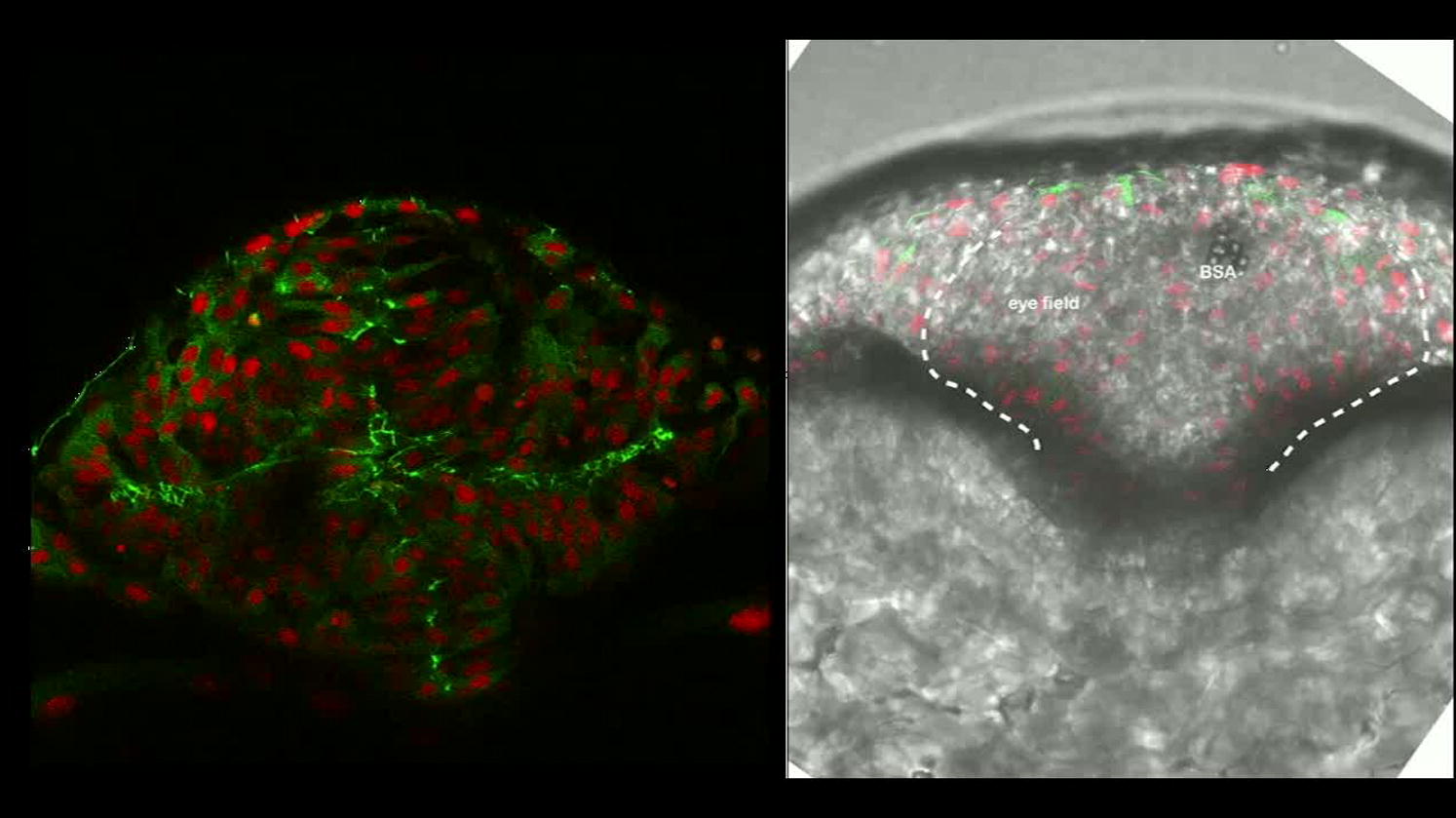

Supplement: Movie S9. Laminin1-Coated Beads Implanted in the Eye Field Promote Cell Polarity Reorganization, whereas BSA-Coated Beads Do Not, Related to Figure 6 — Left: Time-lapse of an embryo in which Laminin1-coated beads (blue patch) have been implanted in the center of the eye field. Eye cells around the beads organize with their apical domains oriented away from the beads. The final time point of the movie is shown at different z levels to fully illustrate the reorganization of the cells. Right: Time-lapse of an embryo in which BSA-coated beads (blue patch) were implanted in the center of the eye field. Unlike Lamin1-coated beads, cells do not reorient their apicobasal polarity in response to the BSA beads. Both movies show a single confocal z section. Interval between frames: 6 min. [file mmc10.jpg]
